# Transcriptome analysis of the ependymal barrier during murine neurocysticercosis

**DOI:** 10.1186/1742-2094-9-141

**Published:** 2012-06-25

**Authors:** Pramod Kumar Mishra, Judy M Teale

**Affiliations:** 1 Department of Microbiology and Immunology, University of Texas Health Science Center, San Antonio, TX, USA; 2 Department of Biology, University of Texas, San Antonio, TX, USA; 3 Blood-Brain Barrier Group, Pennington Biomedical Research Center, Baton Rouge, LA, 70808, USA; 4 Department of Biology, The University of Texas at San Antonio, One UTSA Circle, San Antonio, TX, 78249-1644, USA

## Abstract

**Background:**

Central nervous system (CNS) barriers play a pivotal role in the protection and homeostasis of the CNS by enabling the exchange of metabolites while restricting the entry of xenobiotics, blood cells and blood-borne macromolecules. While the blood–brain barrier and blood-cerebrospinal fluid barrier (CSF) control the interface between the blood and CNS, the ependyma acts as a barrier between the CSF and parenchyma, and regulates hydrocephalic pressure and metabolic toxicity. Neurocysticercosis (NCC) is an infection of the CNS caused by the metacestode (larva) of *Taenia solium* and a major cause of acquired epilepsy worldwide. The common clinical manifestations of NCC are seizures, hydrocephalus and symptoms due to increased intracranial pressure. The majority of the associated pathogenesis is attributed to the immune response against the parasite. The properties of the CNS barriers, including the ependyma, are affected during infection, resulting in disrupted homeostasis and infiltration of leukocytes, which correlates with the pathology and disease symptoms of NCC patients.

**Results:**

In order to characterize the role of the ependymal barrier in the immunopathogenesis of NCC, we isolated ependymal cells using laser capture microdissection from mice infected or mock-infected with the closely related parasite *Mesocestoides corti*, and analyzed the genes that were differentially expressed using microarray analysis. The expression of 382 genes was altered. Immune response-related genes were verified by real-time RT-PCR. Ingenuity Pathway Analysis (IPA) software was used to analyze the biological significance of the differentially expressed genes, and revealed that genes known to participate in innate immune responses, antigen presentation and leukocyte infiltration were affected along with the genes involved in carbohydrate, lipid and small molecule biochemistry. Further, MHC class II molecules and chemokines, including CCL12, were found to be upregulated at the protein level using immunofluorescence microcopy. This is important, because these molecules are members of the most significant pathways by IPA analyses.

**Conclusion:**

Thus, our study indicates that ependymal cells actively express immune mediators and likely contribute to the observed immunopathogenesis during infection. Of particular interest is the major upregulation of antigen presentation pathway-related genes and chemokines/cytokines. This could explain how the ependyma is a prominent source of leukocyte infiltration into ventricles through the disrupted ependymal lining by way of pial vessels present in the internal leptomeninges in murine NCC.

## Introduction

There are different central nervous system (CNS) barriers, which play a critical role in the homeostasis of the CNS [1]. However, CNS infections lead to the dysfunction of these barriers. Compromised barrier properties result in leakage of blood-born cells and molecules, as well as a perturbed homeostasis of ions and macromolecules causing brain pathology [1]. Neurocysticercosis (NCC) is a CNS infection caused by the metacestode larva(e) form of the parasite *Taenia solium* and is characterized by a range of pathological symptoms including epileptic seizures, headaches and hydrocephalus [2]. Recently, using a murine model for NCC, we have reported that the distinct CNS barriers are differentially compromised during parasite infection [2-5].

The CNS barriers include the blood–brain barrier (BBB) comprised of pial and parenchymal vessels present in the leptomeninges and cortex, respectively, and the blood cerebrospinal fluid barrier (BCB) present in the choroid plexus [1,6]. The BBB and BCB prevent circulating blood cells and macromolecules from getting into the CNS as well as selectively regulating the exchange of metabolites between blood and CSF. In addition to these barriers, the lining of the ventricles known as the ependymal layer functions as a third barrier separating the CSF from the CNS tissue/parenchyma [7].

The ependymal barrier is composed of a single layer of uniformly arranged ependymal cells or ependymocytes of epithelial nature [1,7,8]. The barrier properties of ependyma are evident from the presence of molecules known to contribute to intercellular communication, paracellular sealing and adherence of cells such as the gap junction molecule connexin-43 [9,10], the tight junction molecules occludin and ZO-1, and adherens junction molecules such as cadherins, α-catenin and β-catenin [3,11]. Ependymal barrier function is further supported by the presence of enzymes such as glutathione-S-transferase isoforms and monoamine oxidase (MAO), which are known to participate in the metabolism of neuroactive and vasoactive amines enabling the ependyma to act as an amine-barrier system [12-15]. In addition, the presence of electrolyte and water transporters such as aquaporin family member 4 (AQP4) participates in the maintenance of hydrocephalic pressure [16]. The ependyma also appears to play a crucial role in the homeostasis of the CNS by exchanging toxic metabolic byproducts and water between the interstitial fluid of CNS cells and the CSF in ventricles. However, despite the known, critical barrier properties of the ependymal layer, the effect of infection on ependymal cells has been largely understudied.

Previously, we have reported the presence and induction of Toll-like receptors (TLRs) in the ependyma during murine NCC [17]. We have also described leukocyte infiltration through the ependyma [3], indicating the compromised integrity of the ependymal barrier [3,5,17]. We hypothesize that the ependyma can be an immunologically active site that upon activation can produce immune effector molecules that support leukocyte transmigration and the dysfunction of the ependymal barrier. To test this hypothesis, we isolated ependymal cells using Laser Capture Microdissection (LCM) and applied a holistic approach of microarray analyses to analyze potential infection-induced changes in gene expression of the ependymal barrier. Results from the transcriptome analyses of the ependyma indicate that networks of genes related to the immune response, cellular function and maintenance are affected, which likely contribute to the neuropathology of NCC.

## Materials and methods

### Animals

Three- to 5-week-old female BALB/c mice were purchased from the National Cancer Institute program (Bethesda, MD). Experiments were conducted under the guidelines of the IACUC, University of Texas System, the US Department of Agriculture and the National Institutes of Health.

### Parasites and infection

Parasite maintenance and intracranial infection were performed using our protocol developed earlier [18]. *M. corti* metacestodes were maintained by serial intraperitoneal (i.p.) inoculation of 8- to 12-week-old female BALB/c mice. For intracranial inoculations, parasites were aseptically collected from the i.p. cavity of mice that had been infected for about 4–6 months. Harvested parasites were extensively washed in HBSS. Approximately 70 metacestodes were then suspended in 50 μl of HBSS and injected intracranially into 3- to 5-week-old female BALB/c mice using a 1-ml syringe and a 25-gauge needle [18]. The needle was inserted to a 2-mm depth at the junction of the superior sagittal and the transverse sutures. This allows insertion of the needle into a protective cuff avoiding penetration of the brain tissue. Control mice were injected with 50 μl sterile HBSS using the same protocol. Before intracranial inoculation, mice were anesthetized intramuscularly with a 50-μl mixture of ketamine HCL and xylazine (30 mg/ml ketamine and 4 mg/ml xylazine).

### Laser captured microdissection microscopy

Animals were killed 3 weeks after inoculation, which was the peak of inflammation [18]. Before sacrifice, animals were anesthetized with 50 μl of a mixture of ketamine HCL and xylazine as above, and perfused through the left ventricle with 15 ml of cold PBS. Perfused brains were immediately removed, embedded in OCT resin (Sakura, Torrance, CA) and snap frozen in 2-methyl butane (Fisher Scientific, Pittsburgh, PA) contained in liquid nitrogen and stored at −80°C for later use. Then 10-μm-thick horizontal cryosections were serially cut from each brain and placed on polyethylene naphthalate membrane slides (Leica Microsystems, Wetzlar, Germany). The tissue sections were fixed in −20°C acetone for 20 s and kept in dry ice followed by rapid immunofluorescence staining. Briefly, tissues were subjected to 70% ethanol at −20°C for 3 min and washed with PBS. Sections were incubated with rabbit anti-mouse β catenin (Zymed, San Francisco, CA) primary antibody for 5 min followed by three washes for 1 min each. Rhodamine Red-X (RRX)-conjugated goat anti-rabbit secondary antibody (Jackson ImmunoResearch, West Grove, PA) was applied for 3 min to detect primary antibody followed by three washes. RNasin® RNase Inhibitor (Promega, Madison, WI) and DEPC water were used as required to ensure an RNase-free procedure. Then, sections were serially dehydrated in 70% (10 s), 95% (20 s), 100% (3× for 30 s each) and Shandon Histosolve™ Xylene Substitute, an isoalkene hydrocarbon-based solution (Shandon, Pittsburgh, PA) 2× for 2 min each. Dehydrated sections were kept under desiccation until use for LCM. Three animals per group (mock infection and *M. corti* infection) were used to isolate ependymal cells. LCM was performed with a Leica LMD 7000 micro systems (Leica Microsystems, Wetzlar Germany).

### RNA isolation and linear amplification

From LCM-isolated ependymal cells, RNA was extracted with the Pico Pure^TM^ RNA isolation kit (Arcturus Bioscience, Mountain View, CA) according to manufacturer’s protocol. DNase (Qiagen, Valencia, CA) treatment was performed directly within the purification column to remove any possible genomic contamination during the RNA extraction process. The quality of the RNA was inspected with the Agilent 2100 Bioanalzyer and NanoDrop ND-1000. Samples passing quality control assessment were then subjected to linear amplification and subsequently labeled with the NuGEN Ovation Aminoallyl RNA Amplification and Labeling System (NuGEN Technologies, San Carlos, CA) as per manufacturer’s instructions.

### Microarray and data processing

Arrays were printed at the Duke Microarray Facility using the Genomics Solutions OmniGrid 100 Arrayer and mouse genome oligo set (version 4.0). The Mus musculus Operon v4.0 spotted microarray contains 35,852 longmer probes representing 25,000 genes and about 38,000 gene transcripts (Operon Biotechnologies, Huntsville, AL). The amplified and labeled product was hybridized to Mus musculus Operon v4.0 spotted microarray according to the manufacturer’s protocol at 42°C with the MAUI hybridization system (BioMicro Systems, MAUI hybridization System, Salt Lake City, UT). The array was then washed at increasing stringencies and scanned on a GenePix 4000B microarray scanner (Axon Instruments, Foster City, CA). Data processing and statistical analysis with the software Genespring 7.3 (Agilent Technologies, Redwood City, CA) were used to perform data analysis. Intensity-dependent (Lowess) normalization was done on the entire data set. To assess the quality of the data set, a principle component analysis was performed on samples on expression of all genes with mean centering and scaling. Data sets were filtered based on values, and probe sets with background-subtracted intensity of 44 or less were excluded from the analysis. Based on replicates of each condition, pair-wise comparisons were performed on data set samples from mock-infected vs. infected samples. Differentially expressed probe sets were selected based on a volcano plot with a two-fold change and a *p*-value cutoff of 0.05. Differentially expressed genes were then clustered using Average Linkage with Pearson correlation as the similarity measurement. Molecular networks of the selected molecules and specific pathways were analyzed with Ingenuity Pathway Analysis (IPA) software (Agilent Technologies).

### Real-time RT-PCR analysis

RNA obtained from LCM-isolated ependymal cells was subjected to linear amplification by the WT-Ovation™ Pico System (Nugen Technology). The resulting cDNA was loaded onto Taq-Man Low Density Arrays microfluidic cards, catalog number −4342259 (Applied Biosystems, Carlsbad, CA) preloaded with fluorogenic probes and custom-designed primers for genes of interest and the housekeeping genes β-actin, ribosomal 18 S and GAPDH (glyceraldehyde 3-phosphate dehydrogenase) [19]. In some experiments, the commercially available Mouse Immune Array, catalog number 4367786 (Applied Biosystems) was used. The plates were then loaded on an ABI Prism 7900 HT Sequence Detection System (Applied Biosystems). The target expression levels were normalized to levels of the housekeeping genes 18 S, β-actin and GAPDH in the same sample. In addition, real-time RT-PCR to assess the relative expression of CCL12 and CCL7 chemokines in infected samples over mock-infected samples was performed using Sybr Green (Applied Biosystems, CA). Primers for CCL12, CCL7 and 18 S were generated using Primer Express software (Applied Biosystems) as follows: 18 S, forward: CGG-CTA-CCA-CAT-CCA-AGG-AA, reverse: GCT-GGA-ATT-ACC-GCG-GCT; CCL-12, forward: TGC-CTC-CTG-CTC-ATA-GCT-ACC, reverse: ACT-GGC-TGC-TTG-TGA-TTC-TCC; CCL7, forward: GGA-TCT-CTG-CCA-CGC-TTC-TG, reverse: GGC-CCA-CAC-TTG-GAT-GCT. Expression of each specific gene in infected samples over mock was calculated by the 2-^ΔΔCt^ method, and results are represented as ΔΔCt over mock [20].

### Tissue preparation and immunofluorescence microscopy

Tissue preparation and immunofluorescence (IF) staining were performed using our protocol as described previously [21]. Mock-infected and infected brains were harvested as described above. Perfused brains were immediately removed, embedded in OCT resin (Sakura, Torrance, CA) and stored at −80°C. Serial horizontal 10-μm-thick cryosections were placed on Silane-prep slides (Sigma-Aldrich, St. Louis, MO). The slides were air dried overnight and fixed in fresh acetone for 20 s at RT. Acetone-fixed sections were wrapped in aluminum foil and stored at −80°C or processed immediately for IF staining. Briefly, tissues were fixed in −20°C acetone for 10 min and then hydrated in PBS. Non-specific immunoglobulin binding was blocked by 30-min incubation at RT with 10% serum from the same species that the fluorochrome-conjugated antibodies were derived. Sections were incubated for 40 min with primary antibodies diluted in 3% species-specific serum. Sections were washed 7× for 3 min each after incubation with the specified antibodies. Secondary antibodies were incubated for 30 min at RT when necessary. Then, sections were mounted using Fluorsave reagent (Calbiochem, La Jolla, CA) containing 0.3 μM 4′,6′-diamidino-2-phenylindole dilactate (DAPI; Molecular Probes, Eugene, OR). Negative controls using secondary antibodies alone were included in each experiment and found to be negative for staining. Fluorescence was visualized in a Leica microscope (Leica Microsystems, Wetzlar Germany). Images were acquired and processed using IP lab software and Adobe Photoshop CS2 (Adobe, Mountain View, CA). The primary antibodies against CCL12 (catalog n–o. BAF428) and CCL5 (catalog no. AF478) were bought from R&D systems (Minneapolis, MN). Biotinylated MHC II A/D antibody (catalog n–o. 553622) was purchased from BD Pharmingen™ (San Diego, CA). Secondary antibody rabbit anti-goat conjugated with RRX was obtained from Jackson ImmunoResearch (West Grove, MD) and Streptavidin-RRX from Molecular Probes (Invitrogen, CA).

## Results

### Ependymal cell isolation

**Figure 1:**
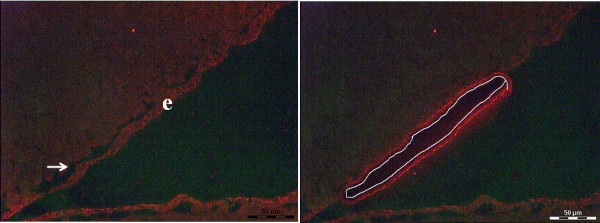
**Immunofluorescence labeling of ependymal cells. (A) **Immunofluorescence labeling of cryostat sections from snap frozen brain tissue for ependymal cells (*e*) using anti- β catenin antibody followed by serial dehydration **(B). **Same section after subjected to LCM-mediated ependymal cells isolation.

LCM was used to select and isolate ependymal cells from mock or parasite-infected mice. A 3-week p.i. time point was used as this is consistently the peak of leukocyte infiltration [3]. In order to do LCM, brain sections were harvested on membrane slides and rapidly labeled for β-catenin by an optimized IF technique for quick labeling of the ependyma followed by dehydration with a xylene substitute as described in Materials and Methods. This causes the ependymal layer to dissociate from closely associated brain parenchyma, thus avoiding unwanted contamination from glial cells (Figure 1). In addition, ependymal cells that line the ventricles can be easily distinguished from the epithelial cells of the choroid plexus (also expressing β-catenin) by anatomical structure and location.

### Assessment of differentially expressed genes in the ependyma resulting from infection

**Figure 2:**
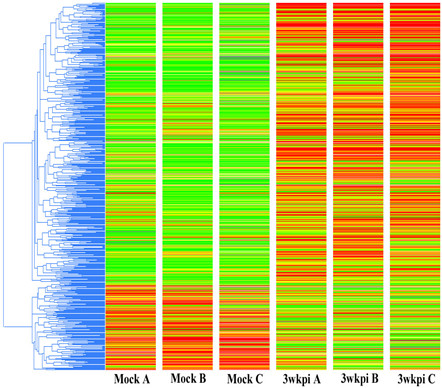
**Differentially expressed probe set in ependymal cells. **Hierarchical cluster analysis of the differentially expressed probe sets by two or more fold; 563 probe sets were significantly affected, (*red* = upregulated, *green * = downregulated). Mock A, B and C represent individual biological samples from infected animals (*n* = 3). Three-week p.i. **A**, **B** and **C **represent individual biological samples from *M. corti*-infected mice (*n* = 3).

To characterize differentially expressed genes, total RNA was isolated from LCM-isolated ependymal cells and subjected to linear amplification. The resulting cDNA was subjected to microarray hybridization on Operon chips containing probe sets representing the mouse genome. Data were processed by Genespring 7.3 software as described in Materials and Methods. A total of 563 probe sets were found to be differentially expressed with more than a two-fold change between mock and infected samples. Using Average Linkage with Pearson correlation as the similarity measurement of gene expression, differentially expressed probe sets were clustered as shown in Figure 2.

Operon chips represent the whole transcriptome derived from the mouse genome depicting both well-characterized genes and uncharacterized genes (Expression Sequence Tags). To identify the genes represented by differentially expressed probe sets, the data generated by Genespring were uploaded into Ingenuity Pathway Analysis software (IPA). Out of 563 probe sets, 400 probes were found annotated and represent 382 genes. Among 382 genes identified based on the common annotation “Ref. Seq,” 301 were upregulated and 81 were downregulated.

The most upregulated genes include ARG1, CD74 (MHC class II invariant chain peptide), CCL8, serum amyloid protein, PSMB9 (proteasomal degradation pathway), SRGN [22], MHC class I and class II haplotype genes, and complement 3 receptor 1 gene, all of which indicate upregulation of immune response genes (Table 1). However, there was no change in the expression of lymphoid markers such as CD4, CD8 and CD19, as well as myeloid cell markers such as CD11b, CD11c and Ly6G between mock-infected and infected mice, suggesting the expression of immune-related genes by ependymal cells. Table 1 also provides the most downregulated genes such as ST18 [23], oncut2 [24,25], ACBD3 [26] and CEP76 [27] encoding for molecules known to participate in cell growth, differentiation and proliferation. A complete list of genes is shown in additional file 1: Table S1.

**Table 1:** **List of the most up- and downregulated (−) genes in ependymal cells from *M. corti***-**infected brain in comparison to mock-infected brain samples**

**Molecules**	**Fold change**	**Molecular function**	**Biological process**
ARG1	142.1	Arginase activity	Arginine and proline metabolism; urea cycle and metabolism of amino groups
CD74	126.8	MHC class II protein binding	Antigen processing and presentation of exogenous peptide antigen via MHC class II
CCL8	83.18	Chemokine activity; heparin binding	Cell-cell signaling; chemotaxis
SAA1	77.66	G-protein coupled receptor binding	Acute-phase response; chemotaxis
PSMB9	74.76	Peptidase activity	Antigen processing and presentation; aprotein polyubiquitination
HLA-C	53.43	Beta-2-microglobulin binding; MHC class I receptor activity	Antigen processing and presentation of peptide antigen via MHC class I
SRGN	53.19	Collagen binding; protein binding	Apoptotic process; biomineral tissue development; blood coagulation; maintenance of granzyme B location in T cell secretory granule
HLA-E	39.98	MHC class I receptor activity	Antigen processing and presentation of peptide antigen via MHC class I
HLA-B	36.35	MHC class I receptor activity	Antigen processing and presentation of peptide antigen via MHC class I
C3AR1	33.81	Complement component C3a receptor activity; G-protein coupled receptor activity;	Chemotaxis; complement receptor-mediated signaling pathway
ST18	−23.92	DNA binding	Regulation of transcription
ONECUT2	−20.08	Sequence-specific DNA binding transcription factor activity	Cell fate commitment; cilium assembly; epithelial cell development
CEP76	−17.01	Protein binding	Regulation of centriole replication
TNNT3	−11.99	Actin binding; calcium- dependent ATPase activity	Muscle contraction; muscle filament sliding; regulation of ATPase activity
AZI2	−6.99	TBK1/IKKi-binding	I-kappaB kinase/NF-kappaB cascade
METTL2B	−6.85	Methyltransferase activity; transferase activity	Methylation
PCF11	−6.8	RNA 3'-end processing	Termination of RNA polymerase II transcription
C20ORF24	−6.67	ND	ND
ACBD3	−6.37	Fatty-acyl-CoA binding	Lipid biosynthetic process; steroid biosynthetic process; transport
SFRS8	−5.65	RNA binding	Negative regulation of nuclear mRNA splicing, regulation of transcription

ND, not determined.

### Biofunctions and networks of differentially expressed genes

To determine the biofunctions associated with infection-induced changes in gene expression, affected gene sets were analyzed using IPA software. IPA uses the knowledge base created from previous findings regarding particular genes and associated functions. During analysis, IPA finds the assigned function for given genes and further categorizes genes with related biological functions into biofunction classes. The top three affected classes for the ependyma were disease and disorder, molecular and cellular function, and physiological system development and function (Table 2). The most significant functions (*p* value, 9.03E-19 to 9.66E-04) within the disease and disorder class were inflammatory response, immunological disease and inflammatory disease. In the molecular and cellular functions class, the most significant functions altered by infection were cellular development, cellular movement, and cellular growth and proliferation (*p* value, 4.49E-14 to 1.61E-03). The most significant functions (*p* value, 1.29E-11 to 1.45E-03) affected in the physiological system, development and function class were hematological system development and function, immune cell trafficking and tissue morphology (Table 2).

**Table 2:** Top biological functions associated with genes differentially expressed in ependymal cells from NCC brain

**Name**	**p-value**	**No. molecules**
**Diseases and disorders**		
Inflammatory response	9.03E-19 - 1.61E-03	109
Immunological disease	2.87E-13 - 1.37E-03	111
Inflammatory disease	1.25E-11 - 9.66E-04	127
Respiratory disease	1.84E-11 - 9.10E-04	51
Connective tissue disorders	2.15E-10 - 7.88E-04	90
**Molecular and cellular functions**		
Cellular development	4.49E-14 - 1.61E-03	97
Cellular movement	5.98E-12 - 1.32E-03	79
Cellular growth and proliferation	2.37E-11 - 1.61E-03	122
Cell-to-cell signaling and interaction	1.36E-10 - 1.61E-03	82
Cell death	2.87E-09 - 1.58E-03	113
**Physiological system development and function**		
Hematological system development and function	1.29E-11 - 1.61E-03	102
Immune cell trafficking	1.29E-11 - 1.61E-03	73
Tissue morphology	9.56E-11 - 1.45E-03	53
Hematopoiesis	4.10E-10 - 1.56E-03	60
Cell-mediated immune response	3.05E-08 - 1.27E-03	47

**Figure 3:**
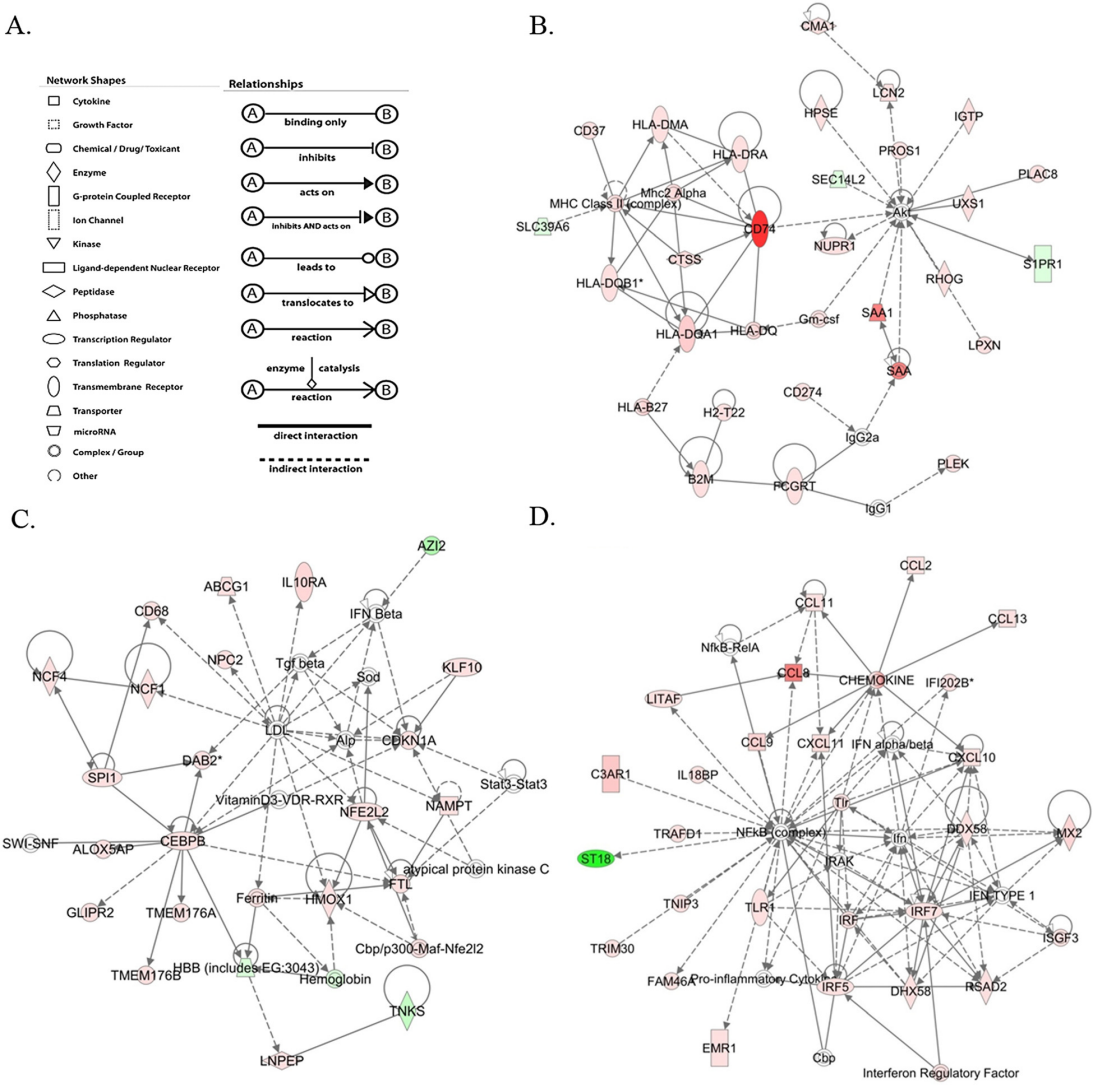
**Schematic representation of the most significant networks predicted by IPA. (A) **Shape and relation legends. **(B) **Antigen presentation, inflammatory response, immunological disease. **(C) **Small molecule biochemistry, carbohydrate metabolism, molecular transport. **(D) **Antimicrobial response, inflammatory response, cell-to-cell signaling interaction. Red shape represents upregulated, green shape represents downregulated, and white boxes represent genes not present in the data sets but relevant to biological pathways and incorporated to generate networks.

To better understand the significance of the differentially expressed genes in relation to each other, we analyzed the biomarkers for networks of genes. Networks are generated based on a random selection of focus genes with maximum connectivity, and several interconnected focus genes are put together as a network in order of high to low scores. The score is calculated through Fisher’s exact test, which represents the ratio between the number of all genes in a given network and the number of focus genes. Based on the focus genes differentially expressed during infection, several networks were identified. Twenty-one networks out of 25 networks yielded a score of more than 3 (Additional file 2: Table S2). The top gene networks included ‘antigen presentation, inflammatory response, immunological disease;’ ‘small molecule biochemistry, carbohydrate metabolism, molecular transport;’ and ‘antimicrobial response, inflammatory response, cell-to-cell signaling and interaction” (Additional file 2: Table S2, Figure 3).

**Table 3:** Verification of infection-induced gene expression in the ependyma by real-time PCR

**Gene symbol**	**Ref. seq.**	**Microarray**	**RT-PCR**		
		**fold change**	ΔΔ **Ct value**	**SE**	***p*-value**
ARG1	NM_007482	142.1	20.67	0.44	<0.001
MRC1	NM_008625	7.893	4.71	0.30	0.001
B2M	NM_009735	19.11	4.49	0.45	<0.01
C3	NM_009778	14.46	4.50	0.26	<0.001
CCL2	NM_011333	3.205	14.10	0.65	<0.001
CCL12	NM_011331	18.84	7.58	0.82	<0.01
CXCL10	NM_021274	2.929	10.68	2.95	<0.05
CXCL11	NM_019494	21.73	10.13	1.60	<0.01
FN1	NM_010233	13.49	5.67	0.78	<0.01
STAT1	NM_009283	6.53	4.07	0.45	<0.01
H2-Ea	NM_010381	16.41	10.54	1.20	<0.01

Genes mentioned in bold red color represent upregulated genes, and genes in bold green color represent downregulated genes. Genes in black color are not affected in the data set but are relevant to the networks. (Description and fold change associated with differentially affected genes are described in Additional file 1: Table S1).

**Figure 4:**
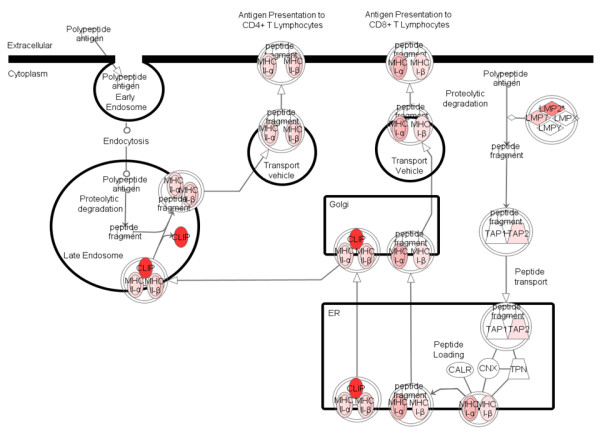
**Schematic representation of the most significantly affected pathway: “Antigen presentation pathway.”** (Red indicates upregulated genes in the ependymal cells from infected brain in comparison to ependymal cells from mock-infected brain samples.).

We further tested for ‘canonical pathways’ within the affected gene networks based on the ratio of molecules known to participate in a given pathway, which were also differentially expressed as a result of infection. The top pathways are shown in Additional file 3: Table S3 and Figure 4. The top affected canonical pathways highlight gene cascades related to the immune response. Of particular interest is the antigen presentation pathway (Figure 4).

Gene expression analysis by real-time PCR using the cDNA linearly amplified from LCM-isolated ependymal cells from mock-infected and infected brain samples. Results are expressed as a ΔΔCt over mock-infected control mice and compared with the microarray outcome

### Verification of infection-induced changes in immune-related gene expression in ependyma

To confirm the altered pattern of gene expression obtained from the microarray analysis, Taqman RT-PCR was performed with amplified cDNA from LCM-isolated ependymal cell RNA for a number of genes (Table 3). Genes were chosen based on the outcome of the network and pathway analysis and with emphasis on the immune response (Figure 3 and Additional file 3: Table S3). All of the genes analyzed were confirmed to be significantly upregulated by RT-PCR and included antigen presentation-related genes β2 microglobulin, MHC II and transcription factor STAT1. Among the chemokines, CCL2, CCL12, CXCL10 and CXCLl1 were upregulated and relevant for trafficking of monocytes and T cells, two leukocyte subsets that are known to traffic through the ependyma during NCC [3]. In addition, CCL5 and CCL7 were shown to be upregulated by RT-PCR (CCL5, 15.5 fold, SE 0.64; CCL7, 8.7 fold, SE 0.74).

### Protein expression of MHC class II and chemokine expression by the ependyma

**Figure 5:**
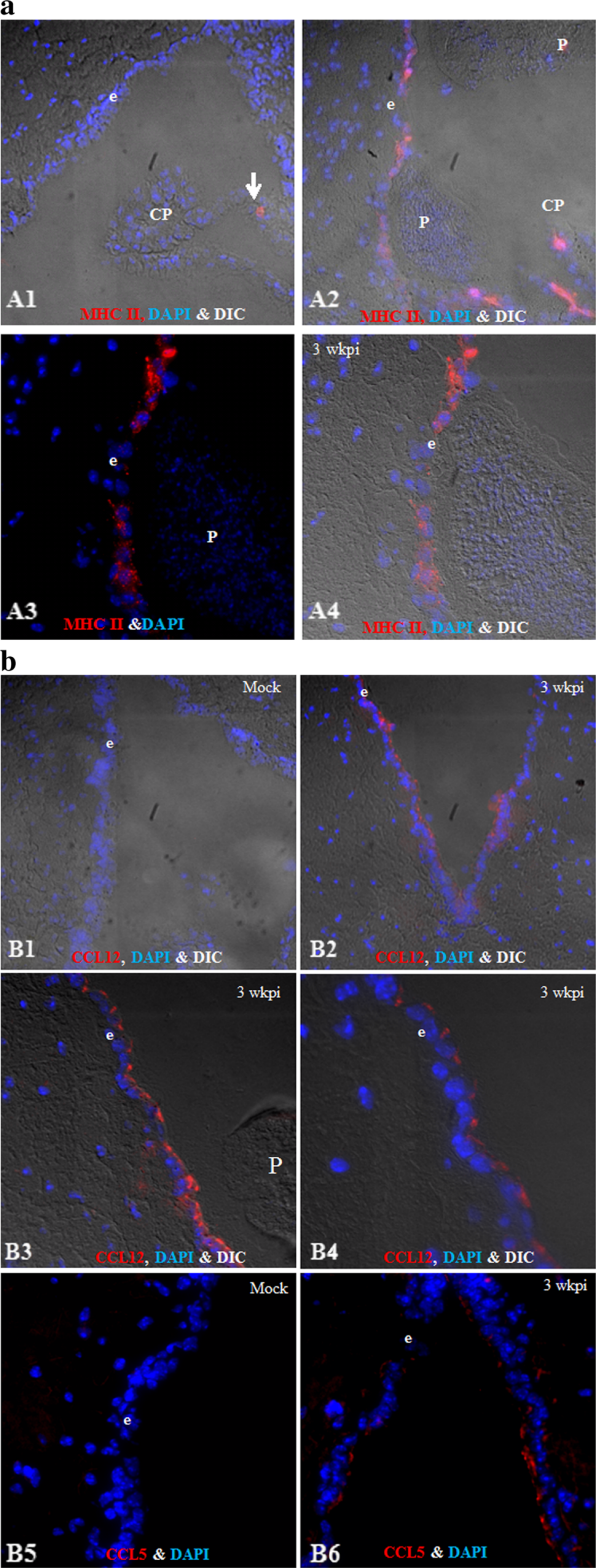
**Schematic representation of the most significantly affected pathway: “Antigen presentation pathway.(a) **MHC II expression in the ependyma, A1. Mock (IF + DIC, 20×), A2. Infected (IF + DIC, 20×). Magnified images (63**×**) showing ependyma during NCC without DIC (A3) and with DIC (A4). **(b) **Chemokine CCL12 (B1-B4) and CCL5 (B5-B6) expression in ependyma, B1. Mock (IF + DIC, 20**×**), B2. Infected (IF + DIC, 20**×**). Magnified images showing CCL12 expression near parasite (B3) and cellular distribution of CCL12 (B4, 63**×**), CCL5 expression in mock (B5, IF, 40**×**) and infected (B6, IF, 40**×**). (*e* = ependyma, *CP* = choroid plexus, *P* = parasite).

Network and pathway analyses showed an increased expression in the ependyma from infected mice of genes responsible for antigen presentation in addition to several chemokines. Brain sections from mock-infected and 3 week p.i. mice were analyzed by IF microscopy to determine if protein expression was similarly increased (Figure 5). In mock-infected samples, MHC II (I-A/I-E) was not detected in the ependyma. However, there were MHC II (I-A/I-E)-positive cells present in the choroid plexuses, presumably circulating leukocytes (Figure 5A1). In infected brains, MHC II (I-A^d^/I-E^d^) expression was observed in ependyma present in the third and fourth ventricles (Figure 5A2-4). Expression of the chemokines CCL12 and CCL5 was undetected in the ependyma from mock-infected animals (Figure 5B1, B5), but at a relatively low basal level of expression in choroid plexuses as well as astrocytic foot processes. After infection, CCL12 (Figure B2-4) and CCL5 (Figure 5, B6) expression was induced in the ependyma in comparison to mock-infected samples. Interestingly, the localization of CCL12 and CCL5 was mainly associated with the basolateral and apical surfaces. Similar results were found for CCL7.

## Discussion

Disruption of the barrier properties of CNS barriers correlates highly with brain pathology [1,28]. In healthy individuals the BBB is controlled in part by endothelial cells that prevent blood cells and blood-borne molecules from entering into the CNS as well as regulating exchange of metabolites. The BCB, which is comprised of the choroid plexuses consisting of modified epithelial cells surrounding fenestrated blood vessels, is a site of CSF production and helps to regulate the exchange of metabolites and provide immune surveillance [29]. During CNS infections, the BBB acts as a route for leukocyte infiltration from blood to the subarachnoid space and parenchyma, while the BCB has been described as a route of leukocyte infiltration from blood to the CSF [6]. However, our prior studies demonstrate that during murine NCC, another route for leukocyte infiltration from blood to ventricles is through a disrupted ependymal layer by way of subependymal pial vessels present in the internal leptomeninges [2,3,5]. Such ependymal disruption correlates with infection-induced increases in MMP8, 3, 12, 2, 9 and 16 in addition to dislocated/rearranged patterns of ependymal junction proteins including occludin, cadherin, catenin α and β [2,3,5]. The ependymal layer consists of specialized epithelial cells that separate the parenchyma and the CSF, and regulate hydrocephalic pressure and metabolic toxicity [7,8]. Of the few studies of the ependyma, most have focused on the transporters or physiological functions of ependyma [30]. Recently, our laboratory and others have shown disease-related induction in ependymal cells of TLR2, 4, 6 and 7 [17,31,32] as well as IL1R [33] and IL18 [34], suggesting that the ependyma contributes to the inflammatory process. It is particularly relevant for NCC since in many clinical cases, as well as in the murine NCC model, parasites can be lodged in ventricles, providing direct access of antigen/PAMPs to ependymal cells [2,35]. The aim of this study was to assess broad-based changes as well as immune-related changes in gene expression of ependymal cells, leading to compromised barrier function and leukocyte infiltration as a result of parasite infection. To date, no comprehensive study has been done with the ependyma, mainly because of the difficulty in isolating ependymal cells.

With the advent of LCM, it is possible to isolate a homogeneous cell population from heterogeneous tissue and monitor such changes. In brain, the ependyma lies between the parenchyma and internal leptomeninges separated by a basement membrane [3]. As mentioned above, optimized dehydration with an isoalkene hydrocarbon-based solution that acts as a substitute for xylene caused the ependymal layer to dissociate from closely associated brain parenchyma and meninges, reducing contamination from other cells (Figure 1). Further, genes highlighted in networks involved in the metabolism and cellular growth as well as in metabolic pathways have been described previously in the ependyma, such as induced expression of IGF1 [8], VIP [8], glucokinase [8], ferric-chelate reductase 1 molecules [36] and adenosine deaminase [37]. In addition, several transporters are affected during infection, which might provide insight into a perturbed homeostasis during NCC because of ependymal cell activation, for example, a reduced expression of the aquaporin family member AQP11. Studies using AQP11-deficient mice have shown an abnormal swelling of epithelial cells in kidneys, leading to cyst formation in proximal tubes and eventually renal failure and death [38]. Similarly, expression of fatty acid-binding protein FABP7, which binds to polyunsaturated fatty acid, correlates with neuroepithelial cell differentiation [39] and ATP1B1, which ensures a proper electrochemical gradient across the plasma membrane, are reduced, among others. In contrast, the expression of the transporter molecule ATP-binding cassette family member, multiple drug resistance protein 3 (ABCC3) is upregulated. ABCC3 is known to be induced as a result of immune activation by the oncostatin-mediated pathway (OSM) [40].

Broad classification of differentially expressed genes in gene networks and related biofunctions indicates that genes involved in antigen recognition, uptake, processing, presentation and immune effector functions are induced. Metacestodes during NCC continuously secrete antigens, particularly glycoconjugates, some of which act as PAMPS [41-46]. The murine NCC model developed in our laboratory using a closely related cestode also shows a similar release pattern of antigenic glycoconjugates. Immune responses vary depending upon the location of metacestodes in the CNS. Metacestodes lodged in ventricles and subarachnoid spaces are known to cause more inflammation, symptoms related with hydrocephalic pressure and associated epileptic seizures [2,35,47]. In a previous study, we showed that parasite-derived antigens are detected in the ventricles and ependymal layer using a monoclonal antibody against a secreted *M. corti* antigen [3]. Possible recognition and phagocytosis are apparent by the induction of pathways such as Fc receptor-mediated phagocytosis and complement.

One of the striking observations is the upregulation of MHC class I and class II genes in ependyma at both the gene expression and protein level. MHC II antigen expression has been detected in non-professional antigen-presenting cells of other immune privileged sites such as the conjunctival epithelium in trachoma [48]. In CNS ventricles, choroid plexus epithelial cells have been shown to have increased expression of MHC I and MHC II antigens upon infection with Theiler’s murine encephalomyelitis virus (TMEV) and spontaneous canine distemper virus encephalitis [49,50]. Further, upon stimulation with IFNγ under *in vitro* conditions, choroid plexus epithelial cells have been shown to express MHC class II molecules and present antigen to T cells [50]. During murine NCC, we have observed the presence of αβ-T cells at 3 weeks p.i. in ventricles [21]. It is possible that antigen availability and the activation state of ependyma influence the trafficking and/or expansion/skewing of T cells in the ventricles. In addition, it might also help in the retention of T cells in ventricles that have migrated through the activated ependyma, as recent reports suggest that antigen presentation upon egress from the BBB helps T cells to stay in the CNS environment [51].

There are several potential mechanisms leading to ependymal cell activation and MHC upregulation evident in the array data. One such gene, STAT1, was verified by RT-PCR [52], which has been shown to regulate the antigen presentation pathway along with IRF1 [53]. Induction of transcription factors STAT1, IRF1, IRF5 and 7 also correlates with our previous finding in which the ependyma was found to express high levels of TLR7 during infection [54] and is in agreement with the study in which IFNγ administration in rats by intravenous infusion led to induction of MHC class II in various cells including the ependyma [55]. Other reports have shown that IFNγ stimulation leads to induction of MHC II in bronchial epithelial cells, and upon engagement with bacterial superantigen, toxic shock syndrome toxin 1 (TSST1) mediates TNFα and IL8 gene expression [56-59].

Our data show that during infection, ependymal cell activation leads to expression of chemokines. Chemokines play a critical role in leukocyte infiltration. Chemokines exert chemotaxis by binding to their respective receptor that leads to downstream signaling events resulting in the tight binding of leukocytes [29]. Our data show that chemokines CCL2, CCL5, CCL7, CCL8, CCL9, CCL6, CCL12, CCL11, CCL13, CXCL10 and CXCL11 are upregulated by infection at the transcript level. Our experiments further indicate the protein localization pattern of CCL12 and CCL5 in apical and baso-lateral surfaces of ependymal cells. Among these chemokines, monocyte chemoattractant protein (MCP) family members CCL2, CCL8, CCL12 and CCL13 are known ligands for CCR2 expressed by monocytes [60]. Studies in the Experimental Autoimmune Encephalitis (EAE) model using *CCR2*^*−/−*^ mice indicate that CCR2 plays a crucial role in recruitment of monocytes, and these mice are resistant to EAE [61]. Among the ligands for CCR2, CCL2 has been shown to be non-redundant as mice lacking CCL2 display a similar clinical phenotype as the *CCR2*^*−/−*^ mice during EAE and are characterized by impaired monocyte recruitment [61]. In addition, CXCL10 and CXCL11 chemokines are recognized by chemokine receptor CXCR3, which is expressed by activated T cells [62,63]. Blockade of CXCL10 with neutralizing antibodies results in reduced numbers of inflammatory T cells and decreased severity of EAE. Similarly, neutralization of CXCR3 reduces CD4^+^ T cell infiltration into the CNS upon intracranial infection with the mouse hepatitis virus (MHV) and mitigates demyelination [64]. CXCR3 has been thought to play a role in retention of CD4^+^ T cells in CNS [62]. Expression of these chemokines by ependymal cells likely attracts leukocytes expressing respective receptors and enhances trafficking of leukocytes into the ventricles originating from pial vessels in the internal leptomeninges.

Taken together, our data suggest that gene expression by ependymal cells is dramatically affected by parasite infection in murine NCC. Although some contamination by extravasating leukocytes present in the ependymal layer cannot be ruled out, it appears to be minimal based on no change in the expression of lymphoid markers such as CD4, CD8 and CD19 as well as myeloid cell markers such as CD11b, CD11c and Ly6G between mock-infected and infected mice. Importantly, the data indicate that the ependyma becomes an immunologically active site. Thus, it appears that the ependyma contributes to the immunopathology associated with NCC and consistent with the ventriculitis associated with this disease [65,66].

## Conclusion

This study provides the first comprehensive gene expression analysis of ependymal cells in a disease condition. Our microarray results derived from ependymal cells establish the biomarkers affected in the ependyma barrier during parasitic infection. Specific pathways indicate that ependymal cells actively express immune mediators and likely contribute to the observed immunopathogenesis during NCC [65-70]. In addition, during CNS infections, the BBB and BCB are described as the main routes of leukocyte infiltration [6]. However, during murine NCC, the ependyma is a prominent source of leukocyte infiltration into the ventricles (blood to the ventricle) [3]. To this end, upregulation of antigen presentation pathway-related genes and chemokines/cytokines are important and provide insight into how ependymal cells could support leukocyte infiltration.

## Competing interests

Both authors declare that they have no competing interests.

## Authors' contributions

Conceived and designed the experiments: PM and JT. Performed the experiments: PM. Analyzed the data: PM and JT. Contributed reagents/materials/analysis tools: JT. Wrote the paper: PM and JT. All authors have read and approved the final manuscript.

## Supplementary Material

Additional file 1 Table S1. Click here for file

Additional file 2 **Table S2. **List of significant networks of gene associated with differentially expressed genes in ependyma from NCC brain. Genes mentioned in bold red color represent upregulated genes, genes in bold green color represent downregulated genes; genes in black color are not affected in the data set but are relevant to the networks. (Description and fold change associated with differentially affected genes are described in Additional file 1: Table S1). Click here for file

Additional file 3 **Table S3. **List of canonical pathways affected in ependyma during NCC (red color represents upregulated genes and green color represents downregulated genes in a given pathway). Click here for file

## References

[B1] Saunders NR Barriers in the brain: a renaissance? Trends Neurosci 2008 31 6 279 286 10.1016/j.tins.2008.03.003 18471905

[B2] Alvarez JI Mesocestoides corti intracranial infection as a murine model for neurocysticercosis Parasitology 2010 137 3 359 372 10.1017/S0031182009991971 20109250

[B3] Alvarez JI Teale JM Differential changes in junctional complex proteins suggest the ependymal lining as the main source of leukocyte infiltration into ventricles in murine neurocysticercosis J Neuroimmunol 2007 187 1–2 102 113 17597230 10.1016/j.jneuroim.2007.05.005 PMC2692657

[B4] Alvarez JI Teale JM Evidence for differential changes of junctional complex proteins in murine neurocysticercosis dependent upon CNS vasculature Brain Res 2007 1169 98 111 17686468 10.1016/j.brainres.2007.07.010 PMC2754301

[B5] Alvarez JI Teale JM Multiple expression of matrix metalloproteinases in murine neurocysticercosis: implications for leukocyte migration through multiple central nervous system barriers Brain Res 2008 1214 145 158 18466882 10.1016/j.brainres.2008.03.036 PMC2517245

[B6] Ransohoff RM Kivisakk P Kidd G Three or more routes for leukocyte migration into the central nervous system Nat Rev Immunol 2003 3 7 569 581 10.1038/nri1130 12876559

[B7] Del Bigio MR The ependyma: a protective barrier between brain and cerebrospinal fluid Glia 1995 14 1 1 13 10.1002/glia.440140102 7615341

[B8] Bruni JE Ependymal development, proliferation, and functions: a review Microsc Res Tech 1998 41 1 2 13 10.1002/(SICI)1097-0029(19980401)41:1<2::AID-JEMT2>3.0.CO;2-Z 9550133

[B9] Yamamoto T Differential anatomical and cellular patterns of connexin43 expression during postnatal development of rat brain Brain Res Dev Brain Res 1992 66 2 165 180 10.1016/0165-3806(92)90077-a 1318799

[B10] Jarvis CR Andrew RD Correlated electrophysiology and morphology of the ependyma in rat hypothalamus J Neurosci 1988 8 10 3691 3702 3193176 10.1523/JNEUROSCI.08-10-03691.1988 PMC6569601

[B11] Lippoldt A Phorbol ester induced changes in tight and adherens junctions in the choroid plexus epithelium and in the ependyma Brain Res 2000 854 1–2 197 206 10784122 10.1016/s0006-8993(99)02355-0

[B12] Abramovitz M Characterization and localization of glutathione-S-transferases in rat brain and binding of hormones, neurotransmitters, and drugs J Neurochem 1988 50 1 50 57 10.1111/j.1471-4159.1988.tb13228.x 2891788

[B13] Carder PJ Glutathione S-transferase in human brain Neuropathol Appl Neurobiol 1990 16 4 293 303 10.1111/j.1365-2990.1990.tb01264.x 2234311

[B14] Saura J Richards JG Mahy N Differential age-related changes of MAO-A and MAO-B in mouse brain and peripheral organs Neurobiol Aging 1994 15 4 399 408 10.1016/0197-4580(94)90071-X 7969716

[B15] Williams D Histochemical characterization of monoamine oxidase in ependyma of rat hypothalamus Histochem J 1979 11 1 83 95 10.1007/BF01041267 429200

[B16] Jung JS Molecular characterization of an aquaporin cDNA from brain: candidate osmoreceptor and regulator of water balance Proc Natl Acad Sci USA 1994 91 26 13052 13056 10.1073/pnas.91.26.13052 7528931 PMC45579

[B17] Mishra BB Mishra PK Teale JM Expression and distribution of Toll-like receptors in the brain during murine neurocysticercosis J Neuroimmunol 2006 181 1–2 46 56 17011049 10.1016/j.jneuroim.2006.07.019 PMC1779953

[B18] Cardona AE Development of an animal model for neurocysticercosis: immune response in the central nervous system is characterized by a predominance of gamma delta T cells J Immunol 1999 162 2 995 1002 9916725

[B19] Mishra BB Gundra UM Teale JM STAT6/mice exhibit decreased cells with alternatively activated macrophage phenotypes and enhanced disease severity in murine neurocysticercosis J Neuroimmunol 2011 232 1–2 26 34 21051093 10.1016/j.jneuroim.2010.09.029 PMC3073530

[B20] Livak KJ Schmittgen TD Analysis of relative gene expression data using real-time quantitative PCR and the 2(−Delta Delta C(T)) Method Methods 2001 25 4 402 408 10.1006/meth.2001.1262 11846609

[B21] Alvarez JI Teale JM Breakdown of the blood brain barrier and blood-cerebrospinal fluid barrier is associated with differential leukocyte migration in distinct compartments of the CNS during the course of murine NCC J Neuroimmunol 2006 173 1–2 45 55 16406118 10.1016/j.jneuroim.2005.11.020

[B22] Toyama-Sorimachi N Widespread expression of chondroitin sulfate-type serglycins with CD44 binding ability in hematopoietic cells J Biol Chem 1997 272 42 26714 26719 10.1074/jbc.272.42.26714 9334256

[B23] Wang S Loss of Myt1 function partially compromises endocrine islet cell differentiation and pancreatic physiological function in the mouse Mech Dev 2007 124 11–12 898 910 17928203 10.1016/j.mod.2007.08.004 PMC2141686

[B24] Vanhorenbeeck V Role of the Onecut transcription factors in pancreas morphogenesis and in pancreatic and enteric endocrine differentiation Dev Biol 2007 305 2 685 694 10.1016/j.ydbio.2007.02.027 17400205

[B25] Margagliotti S The Onecut transcription factors HNF-6/OC-1 and OC-2 regulate early liver expansion by controlling hepatoblast migration Dev Biol 2007 311 2 579 589 10.1016/j.ydbio.2007.09.013 17936262

[B26] Zhou Y The mammalian Golgi regulates numb signaling in asymmetric cell division by releasing ACBD3 during mitosis Cell 2007 129 1 163 178 10.1016/j.cell.2007.02.037 17418793

[B27] Tsang WY Cep76, a centrosomal protein that specifically restrains centriole reduplication Dev Cell 2009 16 5 649 660 10.1016/j.devcel.2009.03.004 19460342 PMC4062978

[B28] Hawkins BT Egleton RD Pathophysiology of the blood–brain barrier: animal models and methods Curr Top Dev Biol 2008 80 277 309 17950377 10.1016/S0070-2153(07)80007-X

[B29] Engelhardt B Sorokin L The blood–brain and the blood-cerebrospinal fluid barriers: function and dysfunction Semin Immunopathol 2009 31 4 497 511 10.1007/s00281-009-0177-0 19779720

[B30] Del Bigio MR Ependymal cells: biology and pathology Acta Neuropathol 2010 119 1 55 73 10.1007/s00401-009-0624-y 20024659

[B31] Letiembre M Innate immune receptor expression in normal brain aging Neuroscience 2007 146 1 248 254 10.1016/j.neuroscience.2007.01.004 17293054

[B32] Xia Y Yamagata K Krukoff TL Differential expression of the CD14/TLR4 complex and inflammatory signaling molecules following i.c.v. administration of LPS Brain Res 2006 1095 1 85 95 10.1016/j.brainres.2006.03.112 16697357

[B33] French RA Expression and localization of p80 and p68 interleukin-1 receptor proteins in the brain of adult mice J Neuroimmunol 1999 93 1–2 194 202 10378883 10.1016/s0165-5728(98)00224-0

[B34] Sugama S Neurons of the superior nucleus of the medial habenula and ependymal cells express IL-18 in rat CNS Brain Res 2002 958 1 1 9 10.1016/S0006-8993(02)03363-2 12468024

[B35] Garcia HH Del Brutto OH Neurocysticercosis: updated concepts about an old disease Lancet Neurol 2005 4 10 653 661 10.1016/S1474-4422(05)70194-0 16168934

[B36] Vargas JD Stromal cell-derived receptor 2 and cytochrome b561 are functional ferric reductases Biochim Biophys Acta 2003 1651 1–2 116 123 14499595 10.1016/s1570-9639(03)00242-5

[B37] Genzen JR Activation of adenosine A2B receptors enhances ciliary beat frequency in mouse lateral ventricle ependymal cells Cerebrospinal Fluid Res 2009 6 15 10.1186/1743-8454-6-15 19922651 PMC2791093

[B38] Morishita Y Disruption of aquaporin-11 produces polycystic kidneys following vacuolization of the proximal tubule Mol Cell Biol 2005 25 17 7770 7779 10.1128/MCB.25.17.7770-7779.2005 16107722 PMC1190286

[B39] Liu RZ Fatty acid binding proteins in brain development and disease Int J Dev Biol 2010 54 8–9 1229 1239 20563994 10.1387/ijdb.092976rl

[B40] Dreuw A Interleukin-6-type cytokines upregulate expression of multidrug resistance-associated proteins in NHEK and dermal fibroblasts J Invest Dermatol 2005 124 1 28 37 10.1111/j.0022-202X.2004.23499.x 15654950

[B41] Obregon-Henao A The role of N-linked carbohydrates in the antigenicity of Taenia solium metacestode glycoproteins of 12, 16 and 18 kD Mol Biochem Parasitol 2001 114 2 209 215 10.1016/S0166-6851(01)00256-0 11378200

[B42] Restrepo BI Analysis of the peripheral immune response in patients with neurocysticercosis: evidence for T cell reactivity to parasite glycoprotein and vesicular fluid antigens Am J Trop Med Hyg 2001 65 4 366 370 11693886 10.4269/ajtmh.2001.65.366

[B43] Lopez-Marin LM Structure and antigenicity of the major glycolipid from Taenia solium cysticerci Mol Biochem Parasitol 2002 119 1 33 42 10.1016/S0166-6851(01)00396-6 11755184

[B44] Obregon-Henao A In situ detection of antigenic glycoproteins in Taenia solium metacestodes J Parasitol 2003 89 4 726 732 10.1645/GE-3046 14533682

[B45] Alvarez JI Rivera J Teale JM Differential release and phagocytosis of tegument glycoconjugates in neurocysticercosis: implications for immune evasion strategies PLoS Negl Trop Dis 2008 2 4 e218 10.1371/journal.pntd.0000218 18398489 PMC2274955

[B46] Estes DM Teale JM Biochemical and functional analysis of extracellular stress proteins of Mesocestoides corti J Immunol 1991 147 11 3926 3934 1940374

[B47] Fleury A Clinical heterogeneity of human neurocysticercosis results from complex interactions among parasite, host and environmental factors Trans R Soc Trop Med Hyg 2010 104 4 243 250 10.1016/j.trstmh.2010.01.005 20116079

[B48] Mabey DC Expression of MHC class II antigens by conjunctival epithelial cells in trachoma: implications concerning the pathogenesis of blinding disease J Clin Pathol 1991 44 4 285 289 10.1136/jcp.44.4.285 2030145 PMC496899

[B49] Alldinger S Up-regulation of major histocompatibility complex class II antigen expression in the central nervous system of dogs with spontaneous canine distemper virus encephalitis Acta Neuropathol 1996 92 3 273 280 10.1007/s004010050518 8870829

[B50] Engelhardt B Wolburg-Buchholz K Wolburg H Involvement of the choroid plexus in central nervous system inflammation Microsc Res Tech 2001 52 1 112 129 10.1002/1097-0029(20010101)52:1<112::AID-JEMT13>3.0.CO;2-5 11135454

[B51] Flugel A Autoaggressive effector T cells in the course of experimental autoimmune encephalomyelitis visualized in the light of two-photon microscopy J Neuroimmunol 2007 191 1–2 86 97 17976745 10.1016/j.jneuroim.2007.09.017

[B52] Uetani K Influenza A virus abrogates IFN-gamma response in respiratory epithelial cells by disruption of the Jak/Stat pathway Eur J Immunol 2008 38 6 1559 1573 10.1002/eji.200737045 18493979

[B53] Reith W LeibundGut-Landmann S Waldburger JM Regulation of MHC class II gene expression by the class II transactivator Nat Rev Immunol 2005 5 10 793 806 10.1038/nri1708 16200082

[B54] Birmachu W Transcriptional networks in plasmacytoid dendritic cells stimulated with synthetic TLR 7 agonists BMC Immunol 2007 8 26 10.1186/1471-2172-8-26 17935622 PMC2175514

[B55] Steiniger B van der Meide PH Rat ependyma and microglia cells express class II MHC antigens after intravenous infusion of recombinant gamma interferon J Neuroimmunol 1988 19 1–2 111 118 3135295 10.1016/0165-5728(88)90040-9

[B56] Nonaka M GM-CSF, IL-8, IL-1R, TNF-alpha R, and HLA-DR in nasal epithelial cells in allergic rhinitis Am J Respir Crit Care Med 1996 153 5 1675 1681 8630619 10.1164/ajrccm.153.5.8630619

[B57] Aubert V Induction of tumor necrosis factor alpha and interleukin-8 gene expression in bronchial epithelial cells by toxic shock syndrome toxin 1 Infect Immun 2000 68 1 120 124 10.1128/IAI.68.1.120-124.2000 10603377 PMC97110

[B58] Kalb TH Evidence for accessory cell function by class II MHC antigen-expressing airway epithelial cells Am J Respir Cell Mol Biol 1991 4 4 320 329 2015098 10.1165/ajrcmb/4.4.320

[B59] Rossi GA Human ciliated bronchial epithelial cells: expression of the HLA-DR antigens and of the HLA-DR alpha gene, modulation of the HLA-DR antigens by gamma-interferon and antigen-presenting function in the mixed leukocyte reaction Am J Respir Cell Mol Biol 1990 3 5 431 439 2145880 10.1165/ajrcmb/3.5.431

[B60] Siebert H The chemokine receptor CCR2 is involved in macrophage recruitment to the injured peripheral nervous system J Neuroimmunol 2000 110 1–2 177 185 11024548 10.1016/s0165-5728(00)00343-x

[B61] Ransohoff RM Chemokines and chemokine receptors: standing at the crossroads of immunobiology and neurobiology Immunity 2009 31 5 711 721 10.1016/j.immuni.2009.09.010 19836265 PMC2787682

[B62] Rebenko-Moll NM Chemokines, mononuclear cells and the nervous system: heaven (or hell) is in the details Curr Opin Immunol 2006 18 6 683 689 10.1016/j.coi.2006.09.005 17010588

[B63] Viola A Luster AD Chemokines and their receptors: drug targets in immunity and inflammation Annu Rev Pharmacol Toxicol 2008 48 171 197 10.1146/annurev.pharmtox.48.121806.154841 17883327

[B64] Stiles LN Differential roles for CXCR3 in CD4+ and CD8+ T cell trafficking following viral infection of the CNS Eur J Immunol 2006 36 3 613 622 10.1002/eji.200535509 16479546

[B65] Cuetter AC Neurocysticercosis: focus on intraventricular disease Clin Infect Dis 1997 24 2 157 164 10.1093/clinids/24.2.157 9114141

[B66] Cuetter AC Andrews RJ Intraventricular neurocysticercosis: 18 consecutive patients and review of the literature Neurosurg Focus 2002 12 6 e5 15926784 10.3171/foc.2002.12.6.6

[B67] Garcia HH New concepts in the diagnosis and management of neurocysticercosis (Taenia solium) Am J Trop Med Hyg 2005 72 1 3 9 15728858

[B68] Cardenas G Subarachnoidal neurocysticercosis non-responsive to cysticidal drugs: a case series BMC Neurol 2010 10 16 10.1186/1471-2377-10-16 20202200 PMC2841125

[B69] Fleury A Subarachnoid basal neurocysticercosis: a focus on the most severe form of the disease Expert Rev Anti Infect Ther 2011 9 1 123 133 10.1586/eri.10.150 21171883

[B70] Couldwell WT Intraventricular neurocysticercosis J Neurosurg 2003 98 3 648 author reply 648–9. 12650445 10.3171/jns.2003.98.3.0648

