# Supplementary material for: Transcriptome analysis of the ependymal barrier during murine neurocysticercosis
Source: J Neuroinflammation. 2012 Jun 25;9:141. doi: 10.1186/1742-2094-9-141 (PMC3527296; doi:10.1186/1742-2094-9-141)
Supplement: Additional file 1 — Table S1. [file 1742-2094-9-141-S1.pdf]

Fold Change	ID	Symbol	Entrez Gene Name
3.919	NM_029639	1600029D21RIK	RIKEN cDNA 1600029D21 gene
10.6	NM_177146	9330175E14RIK	RIKEN cDNA 9330175E14 gene
6.435	NM_213615	A530032D15RIK	RIKEN cDNA A530032D15Rik gene
-3.096	NM_153416	AAAS	achalasia, adrenocortical insufficiency, alacrimia (Allgrove, triple-A)
5.307	XM_358306	ABCC3	ATP-binding cassette, sub-family C (CFTR/MRP), member 3
3.962	NM_009593	ABCG1	ATP-binding cassette, sub-family G (WHITE), member 1
-6.369	NM_133225	ACBD3	acyl-Coenzyme A binding domain containing 3
-2.488	XM_484966	ACBD7	acyl-Coenzyme A binding domain containing 7
-3.774	NM_025790	ACOT13	acyl-CoA thioesterase 13
19.14	NM_007398	ADA	adenosine deaminase
-2.375	NM_009623	ADCY8	adenylate cyclase 8 (brain)
5.224	NM_019467	AIF1	allograft inflammatory factor 1
4.3	NM_172393	AIM1 (includes EG:202)	absent in melanoma 1
-4.717	NM_031185	AKAP12	A kinase (PRKA) anchor protein 12
4.51	NM_009663	ALOX5AP	arachidonate 5-lipoxygenase-activating protein
6.554	NM_008486	ANPEP	alanyl (membrane) aminopeptidase
2.128	NM_013470	ANXA3	annexin A3
6.995	NM_031159	APOBEC1	apolipoprotein B mRNA editing enzyme, catalytic polypeptide 1
7.958	XM_128064	APOL9A (includes EG:223672)	apolipoprotein L 9a
-3.559	NM_175105	AQP11	aquaporin 11
142.1	NM_007482	ARG1	arginase, liver
8.326	NM_146011	ARHGAP9	Rho GTPase activating protein 9
6.104	NM_007486	ARHGDIB	Rho GDP dissociation inhibitor (GDI) beta
2.343	XM_129773	ARPC2	actin related protein 2/3 complex, subunit 2, 34kDa
-4.149	NM_133728	ASNSD1	asparagine synthetase domain containing 1
-2.77	NM_009721	ATP1B1	ATPase, Na+/K+ transporting, beta 1 polypeptide
-6.993	NM_013727	AZI2	5-azacytidine induced 2

19.11	NM_009735	B2M	beta-2-microglobulin
6.63	XM_484142	BAZ1A	bromodomain adjacent to zinc finger domain, 1A
7.021	NM_007535	BCL2A1C	B-cell leukemia/lymphoma 2 related protein A1c
5.164	NM_009763	BST1	bone marrow stromal cell antigen 1
-2.179	NM_138678	BUTR1	butyrophilin related 1
5.041	NM_028732	C10ORF54	chromosome 10 open reading frame 54
3.419	NM_175219	C130026I21RIK	RIKEN cDNA C130026I21 gene
-2.353	NM_199310	C15ORF57	chromosome 15 open reading frame 57
-2.227	NM_175454	C17ORF28	chromosome 17 open reading frame 28
6.214	XM_131779	C1ORF38	chromosome 1 open reading frame 38
3.096	NM_007574	C1QC	complement component 1, q subcomponent, C chain
5.826	NM_023143	C1R	complement component 1, r subcomponent
-6.667	NM_026124	C20ORF24	chromosome 20 open reading frame 24
5.698	NM_177645	C2ORF67	chromosome 2 open reading frame 67
14.46	NM_009778	C3	complement component 3
33.81	NM_009779	C3AR1	complement component 3a receptor 1
6.27	NM_016704	C6	complement component 6
2.137	XM_128587	C6ORF106	chromosome 6 open reading frame 106
3.168	NM_001012667	C8ORF40	chromosome 8 open reading frame 40
3.629	NM_021415	CACNA1H	calcium channel, voltage-dependent, T type, alpha 1H subunit
-5.376	NM_177407	CAMK2A	calcium/calmodulin-dependent protein kinase II alpha
2.78	NM_007599	CAPG	capping protein (actin filament), gelsolin-like
3.104	NM_009807	CASP1	caspase 1, apoptosis-related cysteine peptidase (interleukin 1, beta, convertase)
23.37	NM_009808	CASP12 (includes EG:12364)	caspase 12

4.95	NM_007609	CASP4	caspase 4, apoptosis-related cysteine peptidase
2.347	NM_009811	CASP6	caspase 6, apoptosis-related cysteine peptidase
3.896	NM_009812	CASP8	caspase 8, apoptosis-related cysteine peptidase
9.066	NM_007621	CBR2	carbonyl reductase 2
2.57	NM_172428	CCDC134	coiled-coil domain containing 134
5.606	NM_011330	CCL11	chemokine (C-C motif) ligand 11
3.205	NM_011333	CCL13	chemokine (C-C motif) ligand 13
3.205	NM_011333	CCL2	chemokine (C-C motif) ligand 2
18.84	NM_011331	CCL12	Small inducible cytokine A12 precursor (CCL12) (Monocyte chemotactic protein 5) (MCP-5)
21.54	NM_009139	CCL6	chemokine (C-C motif) ligand 6
83.18	NM_021443	CCL8	chemokine (C-C motif) ligand 8
28.01	NM_011338	CCL9	chemokine (C-C motif) ligand 9
-2.242	NM_007637	CCT5	chaperonin containing TCP1, subunit 5 (epsilon)
5.226	NM_021893	CD274	CD274 molecule
4.623	NM_170758	CD300A	CD300a molecule
5.335	NM_134158	CD300C	CD300c molecule
7.913	NM_007645	CD37	CD37 molecule
7.023	NM_013706	CD52	CD52 molecule
6.886	NM_007651	CD53	CD53 molecule
14.57	NM_009853	CD68	CD68 molecule
126.8	NM_010545	CD74	CD74 molecule, major histocompatibility complex, class II invariant chain
2.522	NM_007669	CDKN1A	cyclin-dependent kinase inhibitor 1A (p21, Cip1)
3.809	NM_009883	CEBPB	CCAAT/enhancer binding protein (C/EBP), beta
-17.007	XM_129027	CEP76	centrosomal protein 76kDa
10.21	NM_008198	CFB	complement factor B
-4.237	NM_009900	CLCN2	chloride channel 2
4.381	NM_010796	CLEC10A	C-type lectin domain family 10, member A
11.53	NM_199311	CLEC4A1	C-type lectin domain family 4, member a1
-2.481	NM_175448	CLVS2	clavesin 2
7.567	NM_010780	CMA1	chymase 1, mast cell
2.771	NM_020557	CMPK2	cytidine monophosphate (UMP-CMP) kinase 2, mitochondrial

2.192	NM_024217	CMTM3	CKLF-like MARVEL transmembrane domain containing 3
-2.874	NM_009953	CRHR2	corticotropin releasing hormone receptor 2
-2.193	NM_009964	CRYAB	crystallin, alpha B
5.564	NM_007781	CSF2RB	colony stimulating factor 2 receptor, beta, low-affinity (granulocyte-macrophage)
3.209	NM_007782	CSF3R	colony stimulating factor 3 receptor (granulocyte)
14.17	NM_007798	CTSB	cathepsin B
5.139	NM_009983	CTSD	cathepsin D
8.947	NM_009984	CTSL2	cathepsin L2
21.16	NM_021281	CTSS	cathepsin S
9.37	NM_022325	CTSZ (includes EG:1522)	cathepsin Z
2.929	NM_021274	CXCL10	chemokine (C-X-C motif) ligand 10
21.73	NM_019494	CXCL11	chemokine (C-X-C motif) ligand 11
2.996	NM_023118	DAB2	disabled homolog 2, mitogen-responsive phosphoprotein (Drosophila)
5.326	NM_172689	DDX58	DEAD (Asp-Glu-Ala-Asp) box polypeptide 58
5.647	XM_358375	DDX60	DEAD (Asp-Glu-Ala-Asp) box polypeptide 60
-2.008	NM_178681	DGKB	diacylglycerol kinase, beta 90kDa
3.12	NM_030150	DHX58	DEXH (Asp-Glu-X-His) box polypeptide 58
-3.03	NM_015789	DKKL1	dickkopf-like 1 (soggy)
2.943	NM_010071	DOK2	docking protein 2, 56kDa
3.482	NM_013739	DOK3	docking protein 3
2.218	NM_007875	DPAGT1	dolichyl-phosphate (UDP-N-acetylglucosamine) N-acetylglucosaminephosphotransferase 1 (GlcNAc-1-P transferase)
3.998	NM_176913	DPEP2	dipeptidase 2
3.025	NM_027878	DRAM1	DNA-damage regulated autophagy modulator 1
-3.413	NM_010080	DSPP	dentin sialophosphoprotein
3.114	NM_019819	DUSP14	dual specificity phosphatase 14
13.17	NM_007895	EAR2	eosinophil-associated, ribonuclease A family, member 2
2.745	NM_025994	EFHD2	EF-hand domain family, member D2
-2.392	NM_153078	EHBP1	EH domain binding protein 1

5.433	NM_133838	EHD4	EH-domain containing 4
13	NM_145158	EMILIN2	elastin microfibril interfacer 2
3.989	NM_010129	EMP3	epithelial membrane protein 3
4.32	NM_010130	EMR1	egf-like module containing, mucin-like, hormone receptor-like 1
4.321	NM_199016	ENPP4	ectonucleotide pyrophosphatase/phosphodiesterase 4 (putative function)
3.59	NM_010161	EVI2A	ecotropic viral integration site 2A
3.317	NM_207214	EXOC5	exocyst complex component 5
-2.309	NM_021272	FABP7	fatty acid binding protein 7, brain
-3.165	NM_028224	FAIM2	Fas apoptotic inhibitory molecule 2
2.232	XM_135029	FAM46A	family with sequence similarity 46, member A
2.396	NM_144846	FAM49B	family with sequence similarity 49, member B
2.29	NM_023229	FASTK	Fas-activated serine/threonine kinase
9.533	NM_010186	FCGR1A	Fc fragment of IgG, high affinity Ia, receptor (CD64)
6.96	NM_010188	FCGR2A	Fc fragment of IgG, low affinity IIa, receptor (CD32)
6.288	NM_010189	FCGRT	Fc fragment of IgG, receptor, transporter, alpha
-2.959	NM_199012	FCHSD2	FCH and double SH3 domains 2
4.039	NM_008013	FGL2	fibrinogen-like 2
13.49	NM_010233	FN1	fibronectin 1
3.102	NM_009146	FRRS1	ferric-chelate reductase 1
3.594	NM_027759	FSIP1	fibrous sheath interacting protein 1
2.939	NM_010240	FTL	ferritin, light polypeptide
3.977	NM_172451	GALNT6	UDP-N-acetyl-alpha-D-galactosamine:polypeptide N-acetylgalactosaminyltransferase 6 (GalNAc-T6)
17.3	NM_025961	GATM	glycine amidinotransferase (L-arginine:glycine amidinotransferase)
10.13	NM_010259	GBP1 (includes EG:14468)	guanylate binding protein 1
10.52	NM_008620	GBP4 (includes EG:17472)	guanylate binding protein 4
3.357	NM_145545	GBP7	guanylate binding protein 7
-2.119	NM_144909	GCKR	glucokinase (hexokinase 4) regulator
2.141	NM_027450	GLIPR2	GLI pathogenesis-related 2
2.406	XM_357752	GM5331	predicted gene 5331
9.939	NM_022024	GMFG	glia maturation factor, gamma

3.246	NM_023121	GNGT2	guanine nucleotide binding protein (G protein), gamma transducing activity polypeptide 2
3.032	NM_029868	GPBP1L1	GC-rich promoter binding protein 1-like 1
2.782	NM_173398	GPR171	G protein-coupled receptor 171
13.63	NM_022320	GPR35	G protein-coupled receptor 35
6.645	NM_134116	GPSM3	G-protein signaling modulator 3 (AGS3-like, C. elegans)
-4.525	NM_008165	GRIA1	glutamate receptor, ionotropic, AMPA 1
2.564	NM_026960	GSDMD	gasdermin D
6.3	NM_010368	GUSB	glucuronidase, beta
14.81	NM_029000	GVIN1	GTPase, very large interferon inducible 1
7.614	NM_023124	H2-Q8	histocompatibility 2, Q region locus 8
8.501	NM_010397	H2-T22	histocompatibility 2, T region locus 22
27.78	NM_010399	H2-T9 (includes EG:15051)	histocompatibility 2, T region locus 9
4.812	NM_010401	HAL	histidine ammonia-lyase
-3.367	NM_008220	HBB (includes EG:3043)	hemoglobin, beta
2.916	NM_001042593	HBS1L	HBS1-like (S. cerevisiae)
5.818	NM_010407	HCK	hemopoietic cell kinase
4.113	NM_010421	HEXA	hexosaminidase A (alpha polypeptide)
5.739	NM_010422	HEXB	hexosaminidase B (beta polypeptide)
2.555	NM_013820	HK2	hexokinase 2
36.35	NM_010394	HLA-B	major histocompatibility complex, class I, B
53.43	NM_010380	HLA-C	major histocompatibility complex, class I, C
4.897	NM_010386	HLA-DMA	major histocompatibility complex, class II, DM alpha
30.6	NM_010378	HLA-DQA1	major histocompatibility complex, class II, DQ alpha 1
15.49	NM_207105	HLA-DQB1	major histocompatibility complex, class II, DQ beta 1
16.41	NM_010381	HLA-DRA	major histocompatibility complex, class II, DR alpha
39.98	NM_010398	HLA-E	major histocompatibility complex, class I, E

5.334	NM_013819	HLA-G	major histocompatibility complex, class I, G
2.185	NM_177338	HMBOX1	homeobox containing 1
4.211	NM_027521	HMHA1	histocompatibility (minor) HA-1
4.796	NM_010442	HMOX1	heme oxygenase (decycling) 1
-2.513	NM_198937	HN1L	hematological and neurological expressed 1-like
2.352	NM_017370	HP	haptoglobin
16.96	NM_152803	HPSE	heparanase
2.949	XM_136108	HUWE1	HECT, UBA and WWE domain containing 1
15.89	NM_008329	IFI16	interferon, gamma-inducible protein 16
11.25	NM_008327	IFI202B	interferon activated gene 202B
6.55	NM_008328	IFI203	interferon activated gene 203
4.182	NM_194067	IFI27	interferon, alpha-inducible protein 27
9.562	NM_023065	IFI30	interferon, gamma-inducible protein 30
12.23	NM_133871	IFI44	interferon-induced protein 44
5.47	NM_031367	IFI44L	interferon-induced protein 44-like
29.79	NM_008330	IFI47	interferon gamma inducible protein 47
3.182	NM_025378	IFITM3	interferon induced transmembrane protein 3 (1-8U)
4.133	NM_184052	IGF1	insulin-like growth factor 1 (somatomedin C)
17.5	NM_018738	IGTP	interferon gamma induced GTPase
23.89	NM_021792	IIGP1	interferon inducible GTPase 1
24.41	NM_008348	IL10RA	interleukin 10 receptor, alpha
10.63	NM_010531	IL18BP	interleukin 18 binding protein
2.787	NM_001025602	IL1RL1	interleukin 1 receptor-like 1
-3.289	NM_139299	IL31RA	interleukin 31 receptor A
4.204	NM_010566	INPP5D	inositol polyphosphate-5-phosphatase, 145kDa
4.178	NM_008390	IRF1	interferon regulatory factor 1
9.868	NM_012057	IRF5	interferon regulatory factor 5
3.108	NM_016850	IRF7	interferon regulatory factor 7
8.085	NM_019440	IRGM2	immunity-related GTPase family M member 2
6.205	NM_015783	ISG15	ISG15 ubiquitin-like modifier
-2.242	NM_026158	ISOC2B	isochorismatase domain containing 2b
2.96	NM_008413	JAK2	Janus kinase 2
-2.506	NM_153572	KATNAL1	katanin p60 subunit A-like 1

-4.274	NM_172615	KIAA0226	KIAA0226
-3.135	NM_172741	KIAA0355	KIAA0355
-3.436	NM_153584	KIAA1370	KIAA1370
3.893	XM_283973	KIAA1797	KIAA1797
4.014	NM_031180	KLB	klotho beta
2.135	NM_013692	KLF10	Kruppel-like factor 10
6.32	NM_010686	LAPTM5	lysosomal protein transmembrane 5
2.141	NM_022964	LAT2	linker for activation of T cells family, member 2
4.759	NM_008491	LCN2	lipocalin 2
4.351	NM_008879	LCP1	lymphocyte cytosolic protein 1 (L-plastin)
2.554	NM_008495	LGALS1	lectin, galactoside-binding, soluble, 1
5.194	NM_010705	LGALS3	lectin, galactoside-binding, soluble, 3
8.717	NM_011150	LGALS3BP	lectin, galactoside-binding, soluble, 3 binding protein
4.473	NM_010708	LGALS9	lectin, galactoside-binding, soluble, 9
3.059	NM_144556	LGI4	leucine-rich repeat LGI family, member 4
4.085	NM_011095	LILRA6	leukocyte immunoglobulin-like receptor, subfamily A (with TM domain), member 6
14	NM_008147	LILRB4	leukocyte immunoglobulin-like receptor, subfamily B (with TM and ITIM domains), member 4
-2.273	NM_181074	LINGO1	leucine rich repeat and Ig domain containing 1
7.901	NM_021460	LIPA	lipase A, lysosomal acid, cholesterol esterase
3.282	NM_019980	LITAF	lipopolysaccharide-induced TNF factor
5.269	NM_172827	LNPEP	leucyl/cystinyl aminopeptidase
3.223	XM_486478	LOC434624	similar to Ferritin light chain 1 (Ferritin L subunit 1)
9.397	NM_134152	LPXN	leupaxin
6.56	NM_153074	LRRC25	leucine rich repeat containing 25
19.78	NM_146069	LRRC33	leucine rich repeat containing 33
11.38	NM_010738	LY6A	lymphocyte antigen 6 complex, locus A
9.951	NM_010741	LY6C1	lymphocyte antigen 6 complex, locus C1
6.026	NM_008529	LY6E	lymphocyte antigen 6 complex, locus E
16.29	NM_017372	LYZ	lysozyme (renal amyloidosis)

8.244	NM_010658	MAFB	v-maf musculoaponeurotic fibrosarcoma oncogene homolog B (avian)
4.029	NM_010764	MAN2B1	mannosidase, alpha, class 2B, member 1
3.232	NM_029103	MANF	mesencephalic astrocyte-derived neurotrophic factor
2.243	NM_011952	MAPK3	mitogen-activated protein kinase 3
-2.415	NM_153058	MAPRE2	microtubule-associated protein, RP/EB family, member 2
-5.348	NM_008590	MEST	mesoderm specific transcript homolog (mouse)
2.8	NM_144797	METRNL	meteorin, glial cell differentiation regulator-like
-6.849	NM_172567	METTL2B	methyltransferase like 2B
20.55	NM_008605	MMP12	matrix metallopeptidase 12 (macrophage elastase)
18.68	XM_134619	MMP27	matrix metallopeptidase 27
12.01	XM_129176	MPEG1	macrophage expressed 1
-3.953	NM_007962	MPZL2	myelin protein zero-like 2
7.893	NM_008625	MRC1L1	mannose receptor, C type 1-like 1
5.107	XM_354744	MRS2	MRS2 magnesium homeostasis factor homolog (S. cerevisiae)
3.823	NM_029499	MS4A4C	membrane-spanning 4-domains, subfamily A, member 4C
13.7	NM_027836	MS4A7	membrane-spanning 4-domains, subfamily A, member 7
8.177	NM_022430	MS4A8B	membrane-spanning 4-domains, subfamily A, member 8B
-2.66	NM_008629	MSI1	musashi homolog 1 (Drosophila)
4.428	NM_010836	MSX3	homeobox, msh-like 3
4.937	NM_026829	MTHFS	5,10-methenyltetrahydrofolate synthetase (5-formyltetrahydrofolate cyclo-ligase)
-2.062	NM_138656	MVD	mevalonate (diphospho) decarboxylase
25.6	NM_010846	MX2	myxovirus (influenza virus) resistance 2 (mouse)
7.626	NM_053214	MYO1F	myosin IF
-2	NM_133939	NAA38	N(alpha)-acetyltransferase 38, NatC auxiliary subunit
2.258	NM_013796	NAGPA	N-acetylglucosamine-1-phosphodiester alpha-N-acetylglucosaminidase

5.754	NM_010871	NAIP	NLR family, apoptosis inhibitory protein
3.008	NM_021524	NAMPT	nicotinamide phosphoribosyltransferase
-2.079	XM_132755	NANOG	Nanog homeobox
16.04	NM_010876	NCF1	neutrophil cytosolic factor 1
3.171	NM_008677	NCF4	neutrophil cytosolic factor 4, 40kDa
2.183	NM_010902	NFE2L2	nuclear factor (erythroid-derived 2)-like 2
-3.636	NM_008694	NGP	neutrophilic granule protein
4.029	NM_023409	NPC2	Niemann-Pick disease, type C2
4.13	NM_199469	NPLOC4	nuclear protein localization 4 homolog (S. cerevisiae)
-4	NM_183261	NR2F2	nuclear receptor subfamily 2, group F, member 2
-2.193	NM_021717	NRIP2	nuclear receptor interacting protein 2
2.165	NM_019738	NUPR1	nuclear protein, transcriptional regulator, 1
3.853	NM_145227	OAS2	2'-5'-oligoadenylate synthetase 2, 69/71kDa
9.382	NM_145209	OASL	2'-5'-oligoadenylate synthetase-like
15.52	NM_011854	OASL2	2'-5' oligoadenylate synthetase-like 2
4.267	NM_133859	OLFML3	olfactomedin-like 3
-2.062	NM_147055	OLFR649	olfactory receptor 649
-20.08	NM_194268	ONECUT2	one cut homeobox 2
7.547	NM_146278	OR4K5	olfactory receptor, family 4, subfamily K, member 5
4.345	NM_011019	OSMR	oncostatin M receptor
4.798	NM_017375	OSTF1	osteoclast stimulating factor 1
12.4	NM_183168	P2RY6	pyrimidinergic receptor P2Y, G-protein coupled, 6
5.203	NM_172893	PARP12	poly (ADP-ribose) polymerase family, member 12
5.213	XM_488522	PARP14	poly (ADP-ribose) polymerase family, member 14
-6.803	NM_029078	PCF11	PCF11, cleavage and polyadenylation factor subunit, homolog (S. cerevisiae)
3.986	NM_011052	PDCD6IP	programmed cell death 6 interacting protein
2.274	NM_027924	PDGFD	platelet derived growth factor D
2.266	NM_009787	PDIA4	protein disulfide isomerase family A, member 4

-2.427	NM_020271	PDXP (includes EG:57026)	pyridoxal (pyridoxine, vitamin B6) phosphatase
2.633	NM_019703	PFKP	phosphofructokinase, platelet
7.39	NM_008842	PIM1	pim-1 oncogene
4.416	NM_133792	PLA2G15	phospholipase A2, group XV
9.395	NM_139198	PLAC8	placenta-specific 8
18.02	NM_019549	PLEK	pleckstrin
2.066	NM_019755	PLP2	proteolipid protein 2 (colonic epithelium-enriched)
2.93	NM_028199	PLXDC1	plexin domain containing 1
-2.494	NM_026164	PNPLA8	patatin-like phospholipase domain containing 8
2.592	NM_027196	POLD4	polymerase (DNA-directed), delta 4
2.688	NM_017401	POLM	polymerase (DNA directed), mu
3.885	NM_011103	PRKCD	protein kinase C, delta
2.683	NM_011173	PROS1	protein S (alpha)
31.33	NM_010724	PSMB8	proteasome (prosome, macropain) subunit, beta type, 8 (large multifunctional peptidase 7)
74.76	NM_013585	PSMB9	proteasome (prosome, macropain) subunit, beta type, 9 (large multifunctional peptidase 2)
3.699	NM_011201	PTPN1	protein tyrosine phosphatase, non-receptor type 1
2.648	NM_175026	PYHIN1 (includes EG:236312)	pyrin and HIN domain family, member 1
-2.513	NM_009001	RAB3A	RAB3A, member RAS oncogene family
2.011	NM_022327	RALB	v-ral simian leukemia viral oncogene homolog B (ras related; GTP binding protein)
2.541	XM_128924	RBM27	RNA binding motif protein 27
12.06	NM_020509	RETNLA	resistin like alpha
2.35	NM_026418	RGS10	regulator of G-protein signaling 10
3.47	NM_172572	RHBDF2	rhomboid 5 homolog 2 (Drosophila)
3.175	NM_019566	RHOG	ras homolog gene family, member G (rho G)
2.376	NM_133955	RHOU	ras homolog gene family, member U
5.332	NM_021384	RSAD2	radical S-adenosyl methionine domain containing 2
-5.814	NM_028351	RSPO3	R-spondin 3 homolog (Xenopus laevis)
-2.012	NM_007901	S1PR1	sphingosine-1-phosphate receptor 1

77.66	NM_011315	SAA1	serum amyloid A1
6.945	NM_028773	SASH3	SAM and SH3 domain containing 3
2.225	NM_009121	SAT1	spermidine/spermine N1-acetyltransferase 1
-2.183	NM_009162	SCG5	secretogranin V (7B2 protein)
3.849	NM_022324	SDF2L1	stromal cell-derived factor 2-like 1
-2.008	NM_144520	SEC14L2	SEC14-like 2 (S. cerevisiae)
7.997	NM_009252	SERPINA3	serpin peptidase inhibitor, clade A (alpha-1 antiproteinase, antitrypsin), member 3
6.262	NM_009251	SERPINA3G	serine (or cysteine) peptidase inhibitor, clade A, member 3G
3.771	NM_175465	SESTD1	SEC14 and spectrin domains 1
-2.268	NM_009144	SFRP2	secreted frizzled-related protein 2
-5.65	NM_172276	SFRS8	splicing factor, arginine/serine-rich 8 (suppressor-of-white-apricot homolog, Drosophila)
-2.053	NM_019812	SIRT1	sirtuin (silent mating type information regulation 2 homolog) 1 (S. cerevisiae)
-3.049	XM_134226	SLC25A42	solute carrier family 25, member 42
3.495	NM_172980	SLC28A2	solute carrier family 28 (sodium-coupled nucleoside transporter), member 2
-2.353	NM_139143	SLC39A6	solute carrier family 39 (zinc transporter), member 6
3.153	NM_011410	SLFN12	schlafen family member 12
-2.674	NM_025690	SLTM	SAFB-like, transcription modulator
2.592	NM_024225	SNX5	sorting nexin 5
3.421	NM_175397	SP110	SP110 nuclear body protein
6.59	NM_011355	SPI1	spleen focus forming virus (SFFV) proviral integration oncogene spi1
4.106	NM_009263	SPP1	secreted phosphoprotein 1
53.19	NM_011157	SRGN	serglycin
-23.923	NM_173868	ST18	suppression of tumorigenicity 18 (breast carcinoma) (zinc finger protein)
3.431	NM_021547	STARD3	StAR-related lipid transfer (START) domain containing 3
6.024	NM_009283	STAT1	signal transducer and activator of transcription 1, 91kDa
2.496	XM_284053	STEAP2	six transmembrane epithelial antigen of the prostate 2

-4.255	NM_008408	STT3A	STT3, subunit of the oligosaccharyltransferase complex, homolog A (S. cerevisiae)
-2.016	NM_009308	SYT4	synaptotagmin IV
2.347	NM_011528	TALDO1	transaldolase 1
2.1	NM_011530	TAP2	transporter 2, ATP-binding cassette, sub-family B (MDR/TAP)
5.115	NM_011539	TBXAS1	thromboxane A synthase 1 (platelet)
3.081	NM_016921	TCIRG1	T-cell, immune regulator 1, ATPase, H+ transporting, lysosomal V0 subunit A3
13.4	NM_031198	TFEC	transcription factor EC
7.529	NM_177911	TGM4	transglutaminase 4 (prostate)
5.107	NM_011593	TIMP1	TIMP metallopeptidase inhibitor 1
7.481	NM_030682	TLR1	toll-like receptor 1
-2.208	NM_080556	TM9SF2	transmembrane 9 superfamily member 2
2.036	NM_025360	TMED3	transmembrane emp24 protein transport domain containing 3
3.309	NM_144830	TMEM106A	transmembrane protein 106A
-2.066	NM_025838	TMEM107	transmembrane protein 107
-4.975	NM_183311	TMEM145	transmembrane protein 145
14.06	XM_128954	TMEM173	transmembrane protein 173
5.432	NM_025326	TMEM176A	transmembrane protein 176A
5.797	NM_023056	TMEM176B	transmembrane protein 176B
2.141	NM_028355	TMEM48	transmembrane protein 48
2.708	NM_145402	TMEM51	transmembrane protein 51
6.726	NM_027206	TNFAIP8L2	tumor necrosis factor, alpha-induced protein 8-like 2
19.51	NM_001001495	TNIP3	TNFAIP3 interacting protein 3
-5.076	NM_175322	TNKS	tankyrase, TRF1-interacting ankyrin-related ADP-ribose polymerase
-11.99	NM_011620	TNNT3	troponin T type 3 (skeletal, fast)
2.373	NM_152800	TOR2A	torsin family 2, member A
16.28	NM_146206	TPCN2	two pore segment channel 2
-2.02	NM_009416	TPM2	tropomyosin 2 (beta)
3.157	NM_172275	TRAFD1	TRAF-type zinc finger domain containing 1
16.27	NM_031254	TREM2	triggering receptor expressed on myeloid cells 2
2.312	NM_011637	TREX1 (includes EG:11277)	three prime repair exonuclease 1
3.743	NM_009277	TRIM21	tripartite motif-containing 21
12.11	NM_009099	TRIM30	tripartite motif-containing 30

-2.174	NM_028063	TRMU	tRNA 5-methylaminomethyl-2-thiouridylate methyltransferase
-3.788	NM_012035	TRPC7	transient receptor potential cation channel, subfamily C, member 7
-2.353	NM_173033	TSTD2	thiosulfate sulfurtransferase (rhodanese)-like domain containing 2
-3.472	XM_127105	TTC7B	tetratricopeptide repeat domain 7B
3.77	NM_181591	TXNDC3	thioredoxin domain containing 3 (spermatozoa)
8.607	NM_019449	UNC93B1	unc-93 homolog B1 (C. elegans)
2.139	NM_026430	UXS1	UDP-glucuronate decarboxylase 1
2.666	NM_012037	VAT1	vesicle amine transport protein 1 homolog (T. californica)
11.6	NM_011691	VAV1	vav 1 guanine nucleotide exchange factor
2.775	NM_011701	VIM	vimentin
2.601	NM_011725	XLR	X-linked lymphocyte-regulated complex
3.904	NM_028864	ZC3HAV1	zinc finger CCCH-type, antiviral 1
-2.342	NM_011743	ZFP106	zinc finger protein 106 homolog (mouse)
-2.584	NM_177643	ZNF281	zinc finger protein 281
7.019	NM_030139	ZNF449	zinc finger protein 449
