# Supplementary material for: Transcriptome analysis of the ependymal barrier during murine neurocysticercosis
Source: J Neuroinflammation. 2012 Jun 25;9:141. doi: 10.1186/1742-2094-9-141 (PMC3527296; doi:10.1186/1742-2094-9-141)
Supplement: Additional file 2 — Table S2. List of significant networks of gene associated with differentially expressed genes in ependyma from NCC brain. Genes mentioned in bold red color represent upregulated genes, genes in bold green color represent downregulated genes; genes in black color are not affected in the data set but are relevant to the networks. (Description and fold change associated with differentially affected genes are described in Additional file 1: Table S1). [file 1742-2094-9-141-S2.doc]

**Table 2. List of significant networks of gene associated with differentially expressed genes in ependyma from NCC brain.**  Genes mentioned in bold red color represent upregulated genes, genes in bold green color represent downregulated genes; genes in black color are not affected in the data set but are relevant to the networks. (Description and fold change associated with differentially affected genes are described in Supplementary Table 1).

| **ID** | **Molecules in Network** | **Score** | **Focus Mol** | **Top Functions** |
| --- | --- | --- | --- | --- |
| 1 | Akt, B2M, CD37, CD74, CD274, CMA1, CTSS, FCGRT, Gm-csf, H2-T22, HLA-B27, HLA-DMA, HLA-DQ, HLA-DQA1, HLA-DQB1, HLA-DRA, HPSE, IgG1, IgG2a, IGTP, LCN2, LPXN, MHC Class II (complex), Mhc2 Alpha, NUPR1, PLAC8, PLEK, PROS1, RHOG, S1PR1, SAA, SAA1, SEC14L2, SLC39A6, UXS1 | 42 | 26 | Antigen Presentation, Inflammatory Response, Immunological Disease |
| 2 | ABCG1, ALOX5AP, Alp, atypical protein kinase C, AZI2, Cbp/p300-Maf-Nfe2l2, CD68, CDKN1A, CEBPB, DAB2, Ferritin, FTL, GLIPR2, HBB (includes EG:3043), Hemoglobin, HMOX1, IFN Beta, IL10RA, KLF10, LDL, LNPEP, NAMPT, NCF1, NCF4, NFE2L2, NPC2, Sod, SPI1, Stat3-Stat3, SWI-SNF, Tgf beta, TMEM176A, TMEM176B, TNKS, VitaminD3-VDR-RXR | 34 | 23 | Small Molecule Biochemistry, Carbohydrate Metabolism, Molecular Transport |
| 3 | AFP, AIF1, C3AR1, CD53, CD68, CD74, DAD1, DGKB, GBP1 (includes EG:14468), H2-Q8, HIF1A, HLA-DR, IFITM3, IFNB1, IL6, KITLG, LAPTM5, LY6E, MANF, MEST, MX2, MYH7, NR3C1, NRIP2, OAS2, PLA2G15, PRKCB, PSMB8, PSMB9, retinoic acid, RNASE1, SP110, SRGN, TBXAS1, TREX1 (includes EG:11277) | 32 | 23 | Cellular Growth and Proliferation, Tissue Development, Immunological Disease |
| 4 | C3AR1, Cbp, CCL8, CCL9, CCL11, CHEMOKINE, CXCL10, CXCL11, DDX58, DHX58, EMR1, FAM46A, IFI202B, Ifn, IFN alpha/beta, IFN TYPE 1, IL18BP, Interferon Regulatory Factor, IRAK, IRF, IRF5, IRF7, ISGF3, LITAF, MX2, NFkB (complex), NfkB-RelA, Pro-inflammatory Cytokine, RSAD2, ST18, Tlr, TLR1, TNIP3, TRAFD1, TRIM30 | 31 | 22 | Antimicrobial Response, Inflammatory Response, Cell-To-Cell Signaling and Interaction |
| 5 | 20s proteasome, Angiotensin II receptor type 1, C/ebp, Cbp/p300, CRHR2, FABP7, GBP1 (includes EG:14468), Growth hormone, Hla-abc, IFI30, IFI44, IFI47, IL31RA, IRF1, IRG, JAK2, LY6E, OAS2, OSMR, p70 S6k, PI3K, Pias, PSMB8, PSMB9, PTPN1, RAB3A, RALB, RETNLA, SP110, SRC, STAT, STAT1, STAT5a/b, TIMP1, Tnf receptor | 31 | 21 | Cellular Development, Cellular Growth and Proliferation, Skeletal and Muscular System Development and Function |
| 6 | Actin, ADA, ANPEP, C6, C8, Calpain, CASP1, CASP4, CASP6, CASP8, CASP12 (includes EG:12364), Caspase, Caspase 3/7, CSF2RB, CTSB, CTSD, Cyclin A, Cyclin E, Cytochrome c, E2f, EMILIN2, FGL2, Focal adhesion kinase, IGF1, Laminin, LGALS3, LY6A, NAIP, PDCD6IP, peptidase, PP2A, PRKCD, Rb, SERPINA3G, TNFAIP8L2 | 31 | 21 | Cell Death, Protein Degradation, Protein Synthesis |
| 7 | ARG1, C3, C1q, C1QC, C1R, CCL2, CFB, Ck2, CRYAB, CTSZ (includes EG:1522), DSPP, DUSP14, Estrogen Receptor, GBP7, HAL, HP, Ifn gamma, Ifnar, IgG, Igm, IL1, Immunoglobulin, LGALS1, Mmp, MMP27, P38 MAPK, SASH3, SAT1, SERPINA3, Stat1 dimer, TH2 Cytokine, Tnf, TNNT3, TRIM21, Trypsin | 28 | 20 | Cellular Movement, Hematological System Development and Function, Immune Cell Trafficking |
| 8 | CD300A, Csf2ra-Csf2rb, CSF3R, CTSL2, DOK3, ERK1/2, Fc receptor, Fcer1, FCGR1A, FCGR1A/2A/3A, FCGR2A, Gm-Csf Receptor, GUSB, HCK, HEXA, HEXB, HLA-C, HLA-E, HLA-G, Ige, IL17R, INPP5D, KIR, KLB, LAT2, LGALS3BP, MHC Class I (complex), MHC CLASS I (family), MHC I-α, NADPH oxidase, P2RY6, Stat1-Stat2, SYK/ZAP, Tap, TAP2 | 27 | 19 | Cell Death, Cellular Growth and Proliferation, Hematological System Development and Function |
| 9 | ABCC3, ABL1, AFP, arginine, CAPG, CBR2, CTSS, CTSZ (includes EG:1522), DKKL1, EPHB2, ERP29, EWSR1, FDFT1, GBP1 (includes EG:14468), GPBP1L1, HPRT1, HRAS, IFI27, IFI44, INS1, Interferon-α Induced, LTBP2, MVD, MX1, MX2, MYL6, MYO1F, PNPLA8, PPARGC1B, PSMB9, SAA1, SLC28A2, TNF, TRPC7, ZFP106 | 27 | 19 | Infection Mechanism, Organismal Injury and Abnormalities, Lipid Metabolism |
| 10 | alcohol group acceptor phosphotransferase, BAZ1A, CCL13, CD300C, Collagen type I, Collagen(s), Fibrinogen, FN1, G protein alphai, GPSM3, HLA-DR, IFI27, IIGP1, IL12 (complex), IL12 (family), IL1RL1, Interferon alpha, ISG15, LILRB4, LY6C1, Mapk, MAPK3, MMP12, MS4A4C, N-cor, NAGPA, p85 (pik3r), PDGF BB, Pi3-kinase, PIM1, PLA2, PLC gamma, Pld, UNC93B1, ZC3HAV1 | 25 | 18 | Tissue Morphology, Organ Development, Renal and Urological System Development and Function |
| 11 | ARG1, Arginase, CLEC10A, DPAGT1, EAR2, ENPP4, GBP4 (includes EG:17472), GSDMD, GUSB, GVIN1, HAVCR2, IFI203, IFI44L, IFNA7, IFNA8, IFNA14, IFNA17, IFNA21, IFNA10 (includes EG:3446), IFNG, IL10RA, IRGM2, LGALS9, MAN2B1, Mannosidase Alpha, MECP2, MHC Class I (complex), MST1R, PCF11, TH1 Cytokine, TH2 Cytokine, TLR1, TLR2, TREM1, TREM2 | 25 | 18 | Cell-To-Cell Signaling and Interaction, Embryonic Development, Hematological System Development and Function |
| 12 | Adaptor protein 2, AFP, C1ORF38, CCL15, CD1B, CD1D, CMPK2, CTNNBL1, DAD1, DDX60, EFHD2, FOXA2, Histone h4, HLA-C, HLA-G, HN1L, HNF4A, IFI30, IFI47, IFNA2, MIR126 (includes EG:406913), NANOG, OASL, ONECUT2, PARP9, RBBP4, RNA polymerase II, Rxr, SGTA, SPP1, SYT4, TCIRG1, TM9SF2, XRCC6, ZNF281 | 23 | 17 | Cell Death, Lipid Metabolism, Small Molecule Biochemistry |
| 13 | ADCY, ADCY8, AKAP12, CACNA1H, Calcineurin protein(s), Calmodulin, CAMK2A, CaMKII, Creb, DOK2, ERK, F Actin, FAIM2, FSH, Gsk3, hCG, Histone h3, HK2, Insulin, LCP1, Lh, MAFB, MAPRE2, PDIA4, Pkc(s), PLC, RGS10, Rock, Rsk, SCG5, SIRT1, TMEM173, TPM2, Vegf, VIM | 22 | 17 | Cellular Development, Cellular Growth and Proliferation, Cellular Movement |
| 14 | ANXA3, ARHGAP9, beta-estradiol, C17ORF28, CCDC134, CD74, CMTM3, Cofilin, CTSD, CXCL11, DDOST, DPEP2, DUSP3, F12, FGF10, IGL@, leukotriene D4, MAPK1, MGST1, P2RY6, PDXP (includes EG:57026), PLXDC1, PPIB, RBBP8, RHBDF2, RhoGap, SAA1, SCG5, SFRP2, SLA, SMAD9, SRGN, UBQLN4, urea, VCAN | 22 | 17 | Cellular Movement, Hematological System Development and Function, Immune Cell Trafficking |
| 15 | 14-3-3, 1600029D21RIK, ACBD3, AIM1 (includes EG:202), amino acids, ATP1B1, ATP1B3, C10ORF54, CCL11, COL7A1, DCT, EMP3, EPHB2, ERBB2, FASTK, FRRS1, Glycogen synthase, GMEB1, GMFG, KIAA1797, MATK, MRC1L1, NEDD9, NFKB2, OASL2, OSMR, PABPC1, PPP2R1A, PRKAR1A, RBM27, SMAD3, SMURF1, STRAP, UBC, VCL | 19 | 15 | Amino Acid Metabolism, Post-Translational Modification, Small Molecule Biochemistry |
| 16 | B2M, C20ORF24, CLCN2, CLVS2, dihydrotestosterone, FGF10, GATM, HINT1, HLA-DQA1, KIAA0355, KPNA3, MIR17 (includes EG:406952), MIR183 (includes EG:406959), NAA38, NOS1, PARP, PARP2, PARP9, PARP12, PARP14, PCNA, PGRMC1, POLD4, POLM, S100A11, SAR1A, SHMT1, TALDO1, TARDBP, TGFB1, TGM4, Tnf, uric acid, VAT1, XRCC1 | 19 | 15 | Cellular Development, Cellular Growth and Proliferation, Cellular Function and Maintenance |
| 17 | ACOT13, ARNT2, BAT3, BST1, CLTCL1, DAD1, DDOST, EHBP1, EHD3, GCKR, GPS2, HBS1L, HMOX2, HNF4A, HSPB3, IL3, IL15, MEN1, MPZL2, MRPL44, MS4A8B, MTHFS, NCOA3, OLFML3, SETDB1, SFRS8, SIM1, SNX5, STT3A, TMEM48, TOR2A, TRAF2, TTR, WASL (includes EG:8976) | 18 | 14 | Cell-To-Cell Signaling and Interaction, Cellular Growth and Proliferation, Hematological System Development and Function |
| 18 | 14-3-3, Ap1, ARHGDIB, ARPC2, CD3, CD52, CD53, Collagen type IV, EHD4, GRIA1, Integrin, JAK, Jnk, Mek, NFAT (complex), Nfat (family), Pdgf, Pdgf Ab, PDGF-AA, PDGFD, PLP2, Rac, Raf, Rap1, Ras, Ras homolog, RHOU, Sapk, Shc, Sos, STARD3, TCR, TREM2, VAV, VAV1 | 12 | 12 | Cell-To-Cell Signaling and Interaction, Cellular Function and Maintenance, Inflammatory Response |
| 19 | APOBEC1, APOBEC2, BTF3, CCT5, DDT, DRAM1, FAM49B, HINT1, HMBOX1, IFI16, IKBKE, IMPDH2, METTL2B, MIR124, MIR146A (includes EG:406938), MIRN341, MSI1, NFYB, NR2F2, NUMB, PDCD5, PFAS, PGRMC1, PRRX2, SNX3, SRP14, TAGLN2, TARS, TBCA, TMED3, TP53, TRMT112, TTC7B, VHL, VMA21 | 12 | 11 | Tumor Morphology, Cell Cycle, Cell-To-Cell Signaling and Interaction |
| 20 | ACAT1, APP, ARF6, BTF3, CASK, CCL6, CLIC1, EXOC5, FCHSD2, FPR2, GANAB, GLUL, HLA-B, IL4, IL22RA2, KIAA1370, LILRA6, LINGO1, LTA4H, MIR292 (includes EG:100049711), NPEPPS, NUDC, PFKP, phosphate, RCC2, SDF2L1, SEC13, SEC23A, SLTM, SRP14, TARS, TFEC, TOMM40, TRIP11, TRMT112 | 12 | 11 | Lipid Metabolism, Small Molecule Biochemistry, Cellular Development |
| 21 | 14-3-3, 20s proteasome, 26s Proteasome, BIRC5, CEP76, CTSD, ES22, GFAP, GNB1, GNGT2, Gngt2-Transducin beta (cone), GRI, Hd-perinuclear inclusions, HISTONE, HTT, HUWE1, LIPA, LYZ, MAGEA11, MAGEA3 (includes EG:4102), MIR184 (includes EG:406960), MPEG1, MS4A7, NPLOC4, OSTF1, PFN2, Pka, PPL, PSMD3, PSMD4, PSMD13, PSMF1, STMN1, TMEM107, Ubiquitin | 12 | 11 | Carbohydrate Metabolism, Small Molecule Biochemistry, Cellular Assembly and Organization |
