# Supplementary material for: Transcriptome analysis of the ependymal barrier during murine neurocysticercosis
Source: J Neuroinflammation. 2012 Jun 25;9:141. doi: 10.1186/1742-2094-9-141 (PMC3527296; doi:10.1186/1742-2094-9-141)
Supplement: Additional file 3 — Table S3. List of canonical pathways affected in ependyma during NCC (red color represents upregulated genes and green color represents downregulated genes in a given pathway). [file 1742-2094-9-141-S3.doc]

**Table 3. List of canonical pathways affected in ependyma during NCC** (red color represents upregulated genes and green color represents downregulated genes in a given pathway)

| Antigen Presentation Pathway | B2M, PSMB9, HLA-DMA, HLA-E, HLA-DRA, HLA-DQA1, HLA-B, PSMB8, CD74, HLA-G, TAP2, HLA-C |
| --- | --- |
| Role of Pattern Recognition Receptors | TLR1, IRF7, C3, OAS2, MAPK3, DDX58, CASP1, C1QC, C3AR1 |
| Fcγ Receptor-mediated Phagocytosis | HMOX1, FCGR2A, PRKCD, ARPC2, MAPK3, HCK, DGKB, VAV1, FCGR1A, INPP5D |
| Complement System | C1R, C3, CFB, C1QC, C6, C3AR1 |
| Sphingosine-1-phosphate Signaling | CASP6, RHOG, MAPK3, CASP1, S1PR1, RHOU, CASP4, ADCY8, PDGFD, CASP8 |
| Acute Phase Response Signaling | C1R, HMOX1, FTL, HP, FN1, C3, MAPK3, CFB, SERPINA3, CEBPB, JAK2, SAA1 |
| GM-CSF Signaling | CSF2RB, CAMK2A, PIM1, MAPK3, HCK, JAK2, STAT1 |
| Systemic Lupus Erythematosus Signaling | HLA-E, FCGR2A, MAPK3, HLA-B, C6, HLA-G, FCGR1A, INPP5D, HLA-C |
| Prolactin Signaling | PRKCD, MAPK3, CEBPB, JAK2, STAT1, IRF1 |
| Interferon Signaling | PSMB8, JAK2, STAT1, IRF1 |
